# Glycophenotyping of mutants of *Lacticaseibacillus paracasei* by lectin microarray

**DOI:** 10.1128/aem.01707-24

**Published:** 2025-07-09

**Authors:** Emi Suzuki, Masaki Serata, Tomoyuki Sako, Sumie Sato, Tohru Iino, Hiroaki Tateno, Jun Hirabayashi

**Affiliations:** 1 Quality Assurance Unit, Yakult Central Institute, Yakult Honsha Co., Ltd. 74051 , Kunitachi-shi, Tokyo, Japan; 2 Basic Research Department, Yakult Central Institute, Yakult Honsha Co., Ltd., Kunitachi-shi, Tokyo, Japan; 3 Retired Researcher, Yakult Central Institute, Yakult Honsha Co., Ltd., Kunitachi-shi, Tokyo, Japan; 4 Cellular and Molecular Biotechnology Research Institute, National Institute of Advanced Industrial Science and Technology (AIST), Tsukuba, Ibaraki, Japan; 5 Institute of Glyco-core Research (iGCORE), Tokai National Higher Education and Research System, Nagoya University 12965, Nagoya, Aichi Prefecture, Japan; Indiana University Bloomington, Bloomington, Indiana, USA

**Keywords:** *Lacticaseibacillus paracasei*, strain Shirota, LcS, cell wall polysaccharide, LCPS-1, LCPS-2, cps1, lectin, rhamnose, microarray

## Abstract

We previously identified a gene cluster of *Lacticaseibacillus paracasei* strain Shirota (YIT 9029) for cell surface long-chain polysaccharides (LCPS-1) biosynthesis, which modulates YIT 9029 activity to induce cytokine production in immune cells, and showed that a lectin microarray can be useful for distinguishing the profile of bacterial cell-surface polysaccharide (PS) structures. Therefore, we isolated disruptive mutant strains of 51 genes predicted to be involved in cell wall PS biosynthesis in YIT 9029. Their binding profiles to lectins in conjunction with their binding abilities to YIT 9029-specific monoclonal antibody (MAb) were compared. The mutants defective in binding to the MAb all had defects within the *cps1* gene cluster. Some mutants were partially bound to MAb, indicating that these genes may influence the synthesis and maturation of LCPS-1. An advanced lectin microarray analyzed the cell surface glycosylation properties of YIT 9029 and its mutants. YIT 9029 bound to a rhamnose (Rha)-specific lectin CSA, and three additional lectins, including an O-glycan binder (rDiscoidin II) and two mannose (Man)-binders (rOrysata and rBanana). Lectin binding specificity was confirmed by a gene complementation assay for the *cps1C* gene and a carbohydrate inhibition assay. When the binding profiles of individual *cps1A* through *cps1J* knockout mutants were compared, typical and specific binding profile patterns were observed, in which some similarities in the functions of each gene could be predicted. In conclusion, the combined use of lectin microarray and a YIT 9029 mutant strain library is a powerful tool for identifying unknown bacterial gene functions related to the cell surface glycome.

**IMPORTANCE:**

Previously, only a limited number of methods have been available for studying mutations in bacterial cell surface polysaccharide structures in relation to gene function. In this study, we focused on the lectin-binding properties of *Lacticaseibacillus paracasei* YIT 9029 (wild type; WT) and investigated the lectin-binding capabilities of 51 cell wall biosynthesis gene disruption strains using lectin microarrays. The results indicated that lectin-binding properties in gene-disrupted strains varied significantly with the presence or absence of long-chain polysaccharides (LCPS-1), ranging from similar to WT to distinctly different. The use of lectin microarrays in conjunction with the YIT 9029 mutant library has been shown to be a highly effective method for identifying the functions of unknown bacterial genes related to cell-surface glycomes. This innovative approach to glycophenotyping allows for the determination of cell wall glycomes associated with bacterial gene functions using lectin microarrays.

## INTRODUCTION

Bacterial cell surface components, such as polysaccharide (PS) capsules, glycoproteins, or glycolipids, comprise a thick peptidoglycan (PG) layer that surrounds the cytoplasmic membrane (1) and are important signaling factors that trigger various host responses, including pathogenesis, host-microbe interaction, immune modulation, and symbiosis (2). Bacterial cell walls have unique structures related to these phenomena (3, 4). Carbohydrates in the form of capsular polysaccharides (CPS) in Gram-positive bacteria and/or lipopolysaccharides in Gram-negative bacteria are the major components on the surface of bacteria. The cell wall of Gram-positive bacteria is a complex assembly of glycopolymers, teichoic acids (TA), and proteins. These molecules possess a thick PG layer surrounding the cytoplasmic membrane (1). Bacterial cell-surface glycans differ substantially between species and strains (4). Moreover, the cell wall PS of lactic acid bacteria (LAB) contains rhamnose (Rha), and the PS structures are highly diverse, depending on the bacterial strain (5, 6).

LAB are industrially important microorganisms for fermented food production. Many LAB strains have been reported to exert beneficial effects through immune modulation of host cells. *Lacticaseibacillus paracasei* (formerly *Lactobacillus casei*) strain Shirota (YIT 9029) exerts immunomodulatory activities *in vitro* (7, 8) and in humans (9–16). In addition, YIT 9029 enhances NK cell activity in healthy volunteers after regular oral feeding with intact cells (17).

YIT 9029 possesses two types of CPS: long-chain polysaccharides (LCPS-1 [18] and LCPS-2 [19], formerly called PS-1 and PS-2, respectively). In previous studies, we first focused on the role of CPS in the immune modulation activities of this bacterium and revealed that certain gene-knockout strains defective in producing LCPS-1 had altered immune modulating activities toward cultured mouse macrophage-like cells (RAW264.7 and J774.1 cells), suggesting that the PS moieties of cell surface structures, including LCPS-1 (20) and LCPS-2 (19), play an important role in YIT 9029 immune modulation activities. To clarify and compare the surface structural characteristics of YIT 9029 and its mutants with different immune modulation activities, we introduced a novel approach for determining the binding profiles of these cells to lectins using liquid-phase lectin microarray technology. From this analysis, we successfully detected structural alterations on the YIT 9029 cell surface in mutants with defects in certain possible glycosylation enzymes (20).

In this study, we aimed to identify the genes of YIT 9029 that are possibly involved in the biosynthesis of genes or influence the structure of the cell surface PS moieties of the cell wall of YIT 9029 by collecting as many genes as possible and by determining the lectin-binding profiles of the gene-disrupted mutant cells in the lectin microarray system. Although databases of human and bacterial glycosyltransferase genes are being developed each year, it is still difficult to determine the role of a particular gene involved in cell wall biosynthesis. Therefore, it would be worthwhile to systematically correlate the changes in the binding profiles of these mutants with lectin probes. We identified all possible genes for the biosynthesis and modification of the cell-wall PS of YIT 9029 based on the similarity of the gene sequences with known proteins, constructed gene knockout mutants, and employed these mutants in the liquid-phase lectin microarray system.

Through this approach using lectin microarray technology, we hoped to gain new insights into the cell surface structures to evaluate other reactivity with YIT 9029-specific monoclonal antibody (MAb) (21) and the characteristics of the bacterial strain YIT 9029.

## RESULTS

### Identification of a cluster of genes associated with the cell wall biosynthesis of YIT 9029

**Fig 1:**
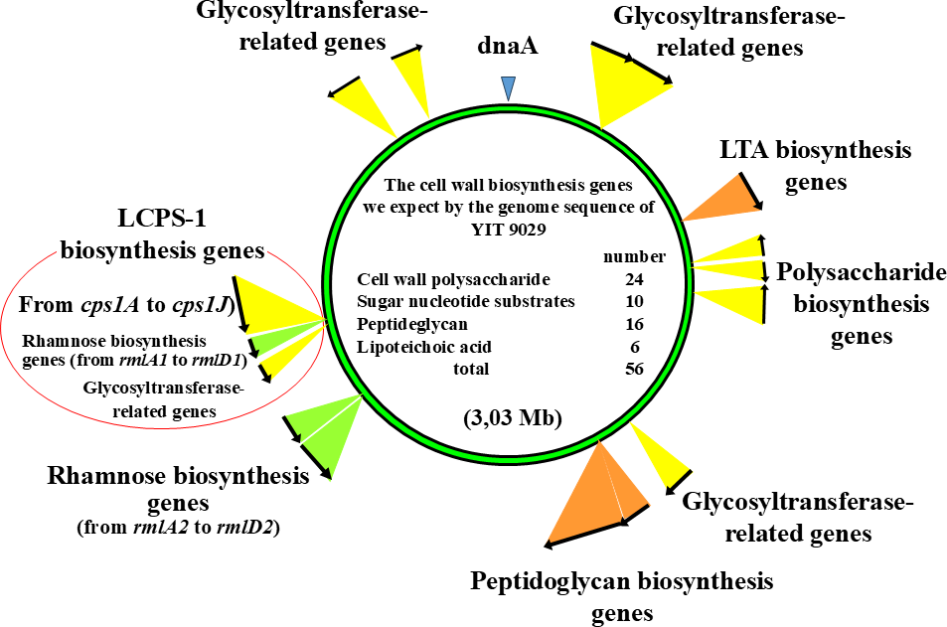
Distribution map of cell wall biosynthesis-related genes in the chromosome of YIT 9029. Fifty-six genes are required for cell wall biosynthesis. Twenty-four, 10, 16, and six genes are required for the biosynthesis of cell wall polysaccharide, sugar nucleotide substrates, peptidoglycan, and lipoteichoic acid, respectively. We succeeded in making 51 gene knockout mutants of YIT 9029. The remaining five were very difficult to isolate.

In a previous study, we identified the biosynthesis genes of LCPS-1; the *cps1* gene cluster consisted of 10 genes designated *cps1A* to *cps1J* (GenBank AB470649) (20). To further analyze the genes responsible for the glycosylation of cell surface molecules of *L. paracase*i, we attempted to identify the genes of YIT 9029 that may participate in the biosynthesis and modification of cell wall PS, TA, and PG, including enzymes for sugar conversion, glycosylation, capsular PS polymerization, and PS repeat unit transporters from the whole genome sequence of YIT 9029. Based on amino acid sequence similarity to known genes of other bacterial strains, we selected 51 candidate genes, including the 10 *cps1* cluster genes described above (Table 1). *RmlA, rmlC, rmlB,* and *rmlD* are the biosynthesis genes of the Rha substance to Glc for cell-wall PSs (22), and two sets of *rml* cluster genes were found on the chromosome of YIT 9029 (Table 1). Unidentified genes located within possible operons of the PS synthetase genes were also included. For glycophenotype analysis, gene-deficient mutants were constructed toward these 51 genes having either deletion (Δ*1932* and Δ*cps1C*) or insertion (marked “Ω”, described in Materials and Methods) within the individual genes (Table S1). Target genes are shown as scattered images in the genome of YIT 9029 (Fig. 1).

**TABLE 1:** Gene library of *L. paracasei* Shirota (YIT 9029) annotated from other lactic acid bacteria and *L. casei* BL23 (23) via amino acid sequence similarities of the cell wall biosynthesis-related genes

*L. paracasei* strain Shirota (YIT 9029)		Annotation of gene before genome information of *L. casei* BL23 was released	Annotation of genome information of *L. casei* BL23 (23)
Target gene			Protein ID
1	*CDS0209*	*Lbul epsM*	CAQ65363.1
2	*CDS0211*	*Llac rgpB*/rhamnosyltransferase	CAQ65364.1
3	*CDS0212*	*Lbul epsH*/glucosyltransferase	CAQ65365.1
4	*CDS0213*	*Lrha epsB*/polysaccharide biosynthesis protein	CAQ65366.1
5	*CDS0214*	*Latilactobacillus sakei* putative autolytic 1,4 beta-MurNAc ase/*L. sakei* putative autolytic 1,4 beta-N-acetylmuramidase	CAQ65367.1
6	*CDS0215*	*Sthe epsI*/glycosyltransferase/similar to *Streptococcus thermophilus EpsI* protein/repeat unit transporter	CAQ65368.1
7	*CDS0216*	*Spne cps19bQ*/probable glycosyl/rhamnosyl transferase	CAQ65369.1
8	*CDS0228*	Transcription repressor/transcription repressor	CAQ65380.1
9	*CDS0229*	*Lbul epsM*/glycosyltransferase/spore coat polysaccharide biosynthesis homolog yveR—*Bacillus subtilis*	CAQ65381.1
10	*CDS0230*	Hypothetical protein	CAQ65382.1
11	*CDS0231*	No hit	CAQ65383.1
12	*CDS0661*	Putative glycosyltransferase	CAQ65850.1
13	*CDS0704*	Dolichol phosphate mannose synthase/glycosyl transferase	CAQ65898.1
14	*CDS0705*	Hypothetical protein ykcB of *B. sub*	CAQ65899.1
15	*CDS0822*	Glycosyltransferase	CAQ66021.1
16	*CDS0823*	Galactosyltransferase homolog	CAQ66022.1
17	*CDS0824*	Conserved membrane protein	CAQ66023.1
18	*CDS0838*	Polysaccharide biosynthesis protein/polysaccharide transporter	CAQ66037.1
19	*CDS0884*	*Lpla tagE2*/glycosyltransferase/poly(glycerol-phosphate) alpha-glucosyltransferase	CAQ66090.1
20	*CDS0885*	*Lpla tagE3*/poly(glycerol-phosphate) alpha-glucosyltransferase	CAQ66091.1
21	*CDS1062*	*Lactobacillus delbrueckii* ArbX protein/glycosyltransferase	CAQ66283.1
22	*CDS1063*	D-galactan O antigen synthesis gene/glycosyl transferase	CAQ66284.1
23	*CDS1064*	Phospho-beta-glycosidase protein	CAQ66285.1
24	*CDS1065*	Alpha-galactosidase	CAQ66286.1
25	*CDS1111*	*Lbul epsJ*/*epsM*, *Llac ycbH*/raffinose-raffinose alpha-galactotransferase	CAQ66336.1
26	*CDS1128*	*Llac ycbB*/glycosyltransferase/stress response protein	CAQ66335.1
27	*CDS1889*	Glucosyl transferase	CAQ67242.1
28	*CDS1892*	Glycosyltransferase	CAQ67245.1
29	*CDS1893*	*rmlD1* (dTDP-dehydrorhamnose reductase)	CAQ67246.1
30	*CDS1894*	*rmlB1* (dTDP-glucose-4,6-dehydratase)	CAQ67247.1
31	*CDS1895*	*rmlC1* (dTDP-dehydrorhamnose 3,5-epimerase)	CAQ67248.1
32	*CDS1896*	*rmlA1* (glucose 1-phosphate thymidyltransferase)	CAQ67249.1
33	*CDS1898*	Glycosyltransferase	CAQ67251.1
34	*CDS1899*	*Llac ycbD*/UDP-glucose 4-epimerase	CAQ67252.1
35	*CDS1926*	*Lrha epsB, Lbul epsD*/capsular polysaccharide biosynthesis	CAQ67280.1
36	*CDS1927*	*Lrha epsA*/transcription regulator/membrane-bound protein	CAQ67281.1
37	*CDS1932*	*rmlD2* (dTDP-dehydrorhamnose reductase)	CAQ67287.1
38	*CDS1933*	*rmlB2* (dTDP-glucose-4,6-dehydratase)	CAQ67288.1
39	*CDS1934*	*rmlC2* (dTDP-dehydrorhamnose 3,5-epimerase)	CAQ67289.1
40	*CDS1935*	*rmlA2* (glucose 1-phosphate thymidyltransferase)	CAQ67290.1
41	*CDS2708*	Glycosyl transferase	CAQ68112.1
42	*cps1A*	*Lbul epsB*/capsular polysaccharide synthesis enzyme/chain length determination	CAQ67302.1
43	*cps1B*	*Lbul epsC*/capsular polysaccharide biosynthesis	CAQ67301.1
44	*cps1C*	*Llac rgpA, Sthe cpsI*/rhamnosyltransferase	CAQ67300.1
45	*cps1D*	*Sthe cpsG*/hexose transferase/glycosyltransferase	CAQ67299.1
46	*cps1E*	*Llac ycbH, Sthe cpsI/epsG*/spore coat polysaccharide biosynthesis protein/galactosyltransferase	CAQ67298.1
47	*cps1F*	Weakly ABC transporter protein	CAQ67297.1
48	*cps1G*	Galactoside acetyltransferase (*lacA*)	CAQ67296.1
49	*cps1H*	*Sthe epsI*/polysaccharide biosynthesis protein/repeat unit transporter	CAQ67295.1
50	*cps1I*	*Spne cps19bQ*/rhamnosyl transferase/glycosyltransferase	CAQ67294.1
51	*cps1J*	*Lbul epsE, Sher epsE*/undecaprenyl-phosphate glycosyl-1-phosphate transferase (*Lactobacillus rhamnosus*)/sugar transferase	CAQ67291.1

### Ability of the mutants to bind to YIT 9029-specific MAb

The YIT 9029-specific MAb recognizes LCPS-1 on the cell surface of YIT 9029. The abilities of the MAb to the mutants were determined by the enzyme-linked immunosorbent assay (ELISA) method (21). Based on the color intensity expressed as actual intensities of absorbance (Table S3), the mutants were largely categorized into three groups: positive, slightly positive, and negative (Table 2). Only seven of the 51 mutants were negative for binding to MAb (21), namely *cps1A, cps1B, cps1C, cps1D, cps1E, cps1G,* and *cps1J,* constituting the *cps1* cluster (20). Eight mutants, including the *cps1F*-deficient mutant, were classified into the slightly positive group and showed decreased antibody reactivity. Thirty-six of 51 mutants were classified as positive, indicating that there was no apparent change in the cell surface PS structure due to these mutations. These mutants are thought to conserve the cell surface structure recognized by the YIT 9029-specific MAb (21). Moreover, the reactivity of YIT 9029-specific MAb (21) in our laboratory collection strains with different antibody reactivity was as follows: YIT 9021 (24), YIT 9022, YIT 9036, and YIT 9037 were slightly positive, positive, negative, and negative, respectively (Table 2).

**TABLE 2:** Reactivity of *L. paracasei* Shirota (YIT 9029) and mutants to *L. paracasei* YIT 9029-specific monoclonal antibody (21)^*a*^

Antibody reactivity	Strain
Positive	YIT 9029 (wild type), Ω*0209*, Ω*0211*, Ω*0214*, Ω*0215*, Ω*0216*, Ω*0228*, Ω*0229*, Ω*0230*, Ω*0231*, Ω*0661*, Ω*0704*, Ω*0705*, Ω*0822*, Ω*0884*, Ω*0885*, Ω*1062*, Ω*1063*, Ω*1064*, Ω*1065*, Ω*1111*, Ω*1128*, Ω*1889*, Ω*1892*, Ω*1893* (*rmlD1*), Ω*1894* (*rmlB1*), Ω*1895* (*rmlC1*), Ω*1896* (*rmlA1*), Ω*1898*, Ω*1899*, Ω*1926*, Δ*1932* (*rmlD2*), Ω*1934* (*rmlC2*), Ω*1935* (*rmlA2*), Ω*1937 (cps1I*), Ω*1938 (cps1H*), Ω*2708*, Δ*cps1C/cps1C,* YIT 9022
Slightly positive	Ω*0212*, Ω*0213*, Ω*0823*, Ω*0824*, Ω*0838*, Ω*1927*, Ω*1933* (*rmlB2*), Ω*1940 (cps1F*), YIT 9021
Negative	Ω*1945 (cps1A*), Ω*1944 (cps1B*), Δ*1943* (*cps1C*), Ω*1942 (cps1D*), Ω*1941 (cps1E*), Ω*1939 (cps1G*), Ω*1936 (cps1J*), YIT 9036, YIT 9037, YIT 0180

^*a*^ The reactivity of the mutants to YIT 9029-specific monoclonal antibody was determined using a sandwich enzyme-linked immunosorbent assay, as described previously (20). The resultant fluorescence intensities of wild type and mutants were classified into three types: “positive” with full to half of the color intensity as that of wild-type YIT 9029, “negative” with very weak or no color, and “slightly positive” with weak or slight color intensity. An absorbance of ≥1.0 was considered positive and <0.25 was considered negative. The absorbance of ≥0.25 but <1.0 was considered slightly positive.

Therefore, the seven genes, *cps1A, cps1B, cps1C, cps1D, cps1E, cps1G,* and *cps1J,* among the 51 genes of YIT 9029, are essential for biosynthesizing LCPS-1.

### Analysis of lectin binding properties of YIT 9029 and influence of *cps1C* gene on lectin binding affinity

**Fig 2:**
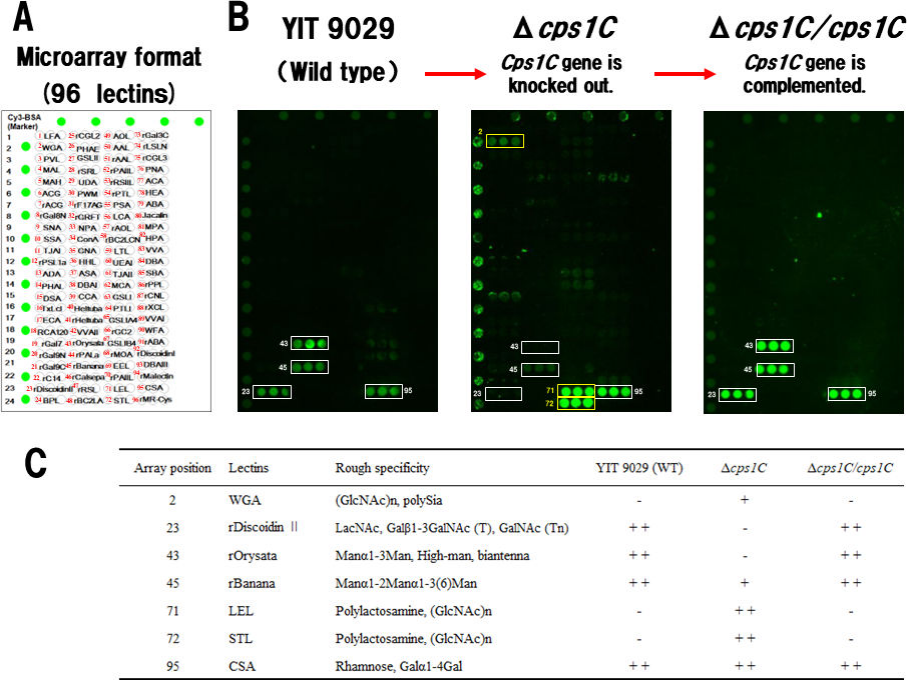
Result of lectin microarray analysis to evaluate the binding properties of YIT 9029 and the influence of the *cps1C* gene on lectin binding affinity. (**A**) Spot pattern of the lectin microarray with 96 lectins immobilized to a glass slide in triplicate (25). (**B**) The lectin binding profiles of YIT 9029 (WT), Δ*cps1C* (*cps1C* gene is knocked out from YIT 9029), and Δ*cps1C/cps1C* (complemented by WT-*cps1C* to Δ*cps1C*) (20). YIT 9029 bound to an O-glycan binder (rDiscoidin II) and two Man binders (rOrysata, rBanana) besides a Rha-binder CSA (formerly CSL) (left). White frames indicate four types of lectins bound to WT-YIT 9029. Among the four lectins bound to YIT 9029, Δ*cps1C* did not bind to rDiscoidin II, Orysata, and rBanana, whereas it bound to CSA. On the other hand, Δ*cps1C* strongly bound to GlcNAc-binders WGA, LEL, and STL, but did not bind to WT-YIT 9029. Yellow frames indicate three lectins specifically bound by Δ*cps1C* (middle). The lectin binding profile of Δ*cps1C/cps1C* was completely recovered to that of YIT 9029 by complementing the WT-*cps1C* gene into Δ*cps1C* (right). (**C**) Summary of the results. Each mark indicates binding affinity to lectins; ++ indicates strong, + indicates weak, and – indicates not binding. YIT 9029, *Lacticaseibacillus paracasei* strain Shirota; WT, wild type; Man, mannose; Rha, rhamnose.

We used a newly improved lectin microarray comprising 96 lectins, including 51 additional lectins (25). The lectin microarray format is shown in Fig. 2A, and the glycan-binding specificities of the lectins used in this study are listed in Table S4. YIT 9029 (WT) showed the affinity to an O-glycan binder (rDiscoidin II), two Man binders (rOrysata and rBanana), and a Rha-binder CSA (formerly CSL) (Fig. 2B, left) (26–32).

Next, the lectin binding profile of Δ*cps1C* deficient in LCPS-1 (20) was different from that of WT; the binding signal to rDiscoidin II and rOrysata disappeared, and that to rBanana was reduced, whereas the binding to CSA was kept. Moreover, Δ*cps1C* strongly bound to WGA, LEL, and STL, to all of which YIT 9029 did not bind (Fig. 2B, middle) (33–35). WGA, LEL, and STL are all GlcNAc-binders and often show specific preferences for polySia and polylactosamine structures. Other lectins to which Δ*cps1C* weakly bound will be described later.

On the other hand, the lectin-binding profile of Δ*cps1C/cps1C,* a derivative of Δ*cps1C* complemented in *trans* by the WT-*cps1C* gene (20), was the same as that of the WT (Fig. 2B, right). The results are summarized in Fig. 2C. These data show that the lectin-binding profile of YIT 9029 is strictly controlled by *cps1C*, a key gene in the biosynthesis of LCPS-1 (20).

YIT 9029 binds to three additional lectins, rDiscoidin II, rOrysata, and rBanana, in addition to CSA (26–32). Moreover, these data indicate that the binding patterns of lectins to bacterial cells are closely associated with the gene functions involved and reflect the cell surface glycosylation profile.

### Carbohydrate inhibition assay

The binding of lectins to YIT 9029 was predicted to be mediated by the affinity of lectins for the carbohydrate moiety of cell surface structures. To speculate and clarify the affinity points for rDiscoidin II, rOrysata, rBanana, and CSA on YIT 9029 (Fig. 2B, left), we attempted to detect possible interference of lectin binding by simple saccharides. We chose Gal, Glu, Lac, Man, Rha, Suc, and Fuc as inhibitors because each lectin bound to YIT 9029 is known to have an affinity for some glycoproteins containing some of these saccharides (36).

**Fig 3:**
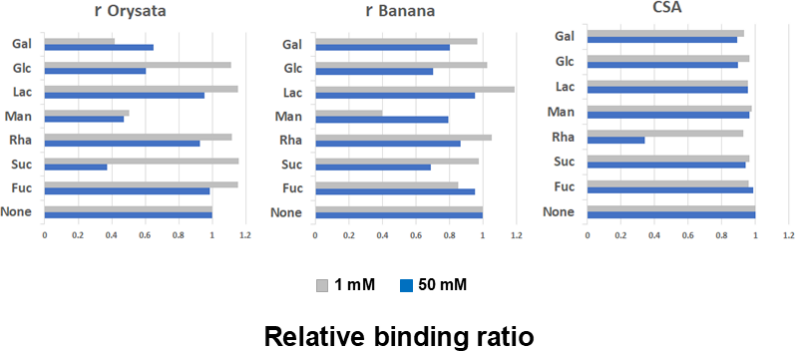
Carbohydrate inhibition assay. D-galactopyranose (Gal), D-glucopyranose (Glc), D-galactosylpyranosyl-(β1→4)-D-Glc (Lac), D-mannopyranose (Man), L-rhamnopyranose (Rha), D-fructofuranosyl-(2↔1)-D-glucopyranoside (Suc), or (3S,4R,5S,6S)-6-methyltetrahydro-2H-pyran-2,3,4,5-tetraol (Fuc) were added in each well at a concentration of 1 mM (above) or 50 mM (below) with YIT 9029 cells (2 × 10^9^ cells/well) labeled with SYTOX orange. None means a control assay in which no carbohydrate is added to the reaction between lectins and YIT 9029. The efficiency of carbohydrate inhibition is shown as the ratio of the fluorescence intensity with carbohydrate against that of the control with no added carbohydrate. The binding of YIT 9029 to rOrysata was inhibited by Gal, Glc, Man, and Suc (left), and that to rBanana and CSA was specifically inhibited by Man and Rha, respectively (middle and right).

The binding affinity of YIT 9029 to rOrysata was inhibited by the addition of 1 mM Gal and Man, and 50 mM Glc, Man, and Suc (Fig. 3, left). Similarly, rBanana was inhibited by the addition of 1 mM Man (Fig. 3, middle). The binding affinity of YIT 9029 to CSA was inhibited by the addition of 50 mM Rha (Fig. 3, right). The binding affinity of YIT 9029 to rDiscoidin II was not inhibited by any saccharides used in this study (data not shown). Man competitively inhibited the binding to rOrysata and rBanana, whereas Rha competitively inhibited the binding to CSA (Fig. 3). These results were consistent with the lectin binding specificities of rOrysata, rBanana, and CSA (26–32).

### Effect of *cps1* cluster genes for LCPS-1 biosynthesis on lectin binding affinity

**Fig 4:**
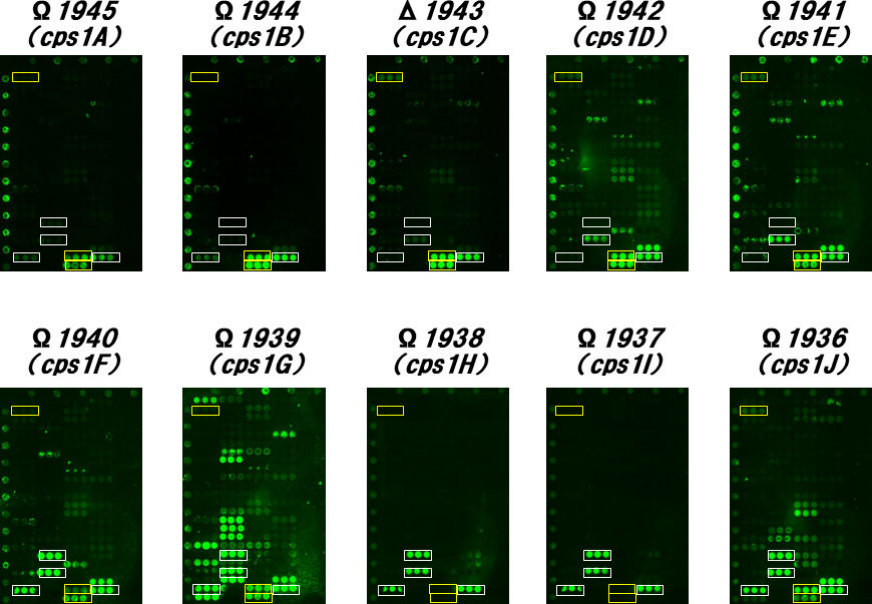
Effect of *cps1* cluster genes essential for LCPS-1 biosynthesis on lectin binding affinity. White frames indicate four lectins, rDiscoidin II, rOrysata, rBanana, and CSA, to which *L. paracasei* strain Sirota (YIT 9029) has specific affinities. Yellow frames indicate three lectins, WGA, LEL, and STL, to which Δ*cps1C* has specific affinities. The lectin binding profiles of Ω*cps1A*, Ω*cps1B*, and Δ*cps1C*; Ω*cps1D* and Ω*cps1E*; Ω*cps1F* and Ω*cps1J*; Ω*cps1H* and Ω*cps1I*, respectively, were well matched. The lectin binding profile of Ω*1938* (*cps1H*) and Ω*1937* (*cps1I*) was completely different from the other eight mutants of disrupted *cps1* cluster genes and similar to that of WT (YIT 9029) (Fig. 2B, left). LCPS-1, long-chain polysaccharide; WT, wild type. The result of Δ*cps1C* is the same as that in the middle of Fig. 2B. Here, the result of Δ*cps1C* is duplicated for comparison of all 10 *cps1* genes involved in LCPS-1 biosynthesis.

To analyze the effects of the genes responsible for biosynthesis of the cell wall polysaccharides of YIT 9029, we first determined the lectin-binding profiles of the gene knockout mutants within the *cps1* gene cluster, which are essential for LCPS-1 biosynthesis using microarray technology (Fig. 2A). The lectin-binding profiles of the 10 knockout mutants from *cps1A* (*CDS1945*) to *cps1J* (*CDS1936*) were unique to one another (Fig. 4). All these mutants, except for Ω*1938* (*cps1H*) and Ω*1937* (*cps1I*), gained the abilities to bind to LEL and STL (34). The binding profiles of Ω*1938* (*cps1H*) and Ω*1937* (*cps1I*) were similar to that of WT (YIT 9029) (Fig. 2B, left). Considering the fact that YIT 9029-specific MAb (21) can bind to Ω*1938* (*cps1H*) and Ω*1937* (*cps1I*) (Table 2) and the previous report (20), it was concluded that the LCPS-1 structure (18) did not change in these two mutants.

The binding profiles of the mutants Ω*1945* (*cps1A*), Ω*1944* (*cps1B*), and Δ*1943* (*cps1C*) were similar to one another because they lost the ability to bind to rDiscoidin II and rOrysata and almost lost the ability to bind to rBanana (26–30). Moreover, the binding profiles of the mutants Ω*1942 (cps1D*) and Ω*1941* (*cps1E*) were similar to each other due to the loss of binding to LEL and rBanana and gain of binding to rMalectin (29, 30, 35). In addition, these mutants showed affinity for some common additional lectins such as rF17AG, rAOL, and TJAII. The mutants Ω*1940* (*cps1F*) and Ω*1936* (*cps1J*) had similar binding profiles by gaining binding ability to LEL, STL, and rMalectin, while maintaining the ability to bind to four lectins to which YIT 9029 binds. However, it is obvious that the structural change in Ω*1940* (*cps1F*) is different from that in Ω*1936,* because Ω*1940* is partially bound to the YIT 9029-specific MAb (21), while Ω*1936* is not. The mutant Ω*1940* had a unique lectin binding profile among the *cps1* cluster genes knockout mutants (20), showing multiple lectin binding capacities.

In contrast, the lectin-binding affinities of Ω*cps1A*, Ω*cps1B*, Δ*cps1C*, Ω*cps1D*, Ω*cps1E*, Ω*cps1F*, and Ω*cps1J* were partially common. These eight mutants were negative or slightly positive for the YIT 9029-specific MAb (21) (Table 2). On the other hand, for three lectins, WGA, LEL, and STL to which Δ*cps1C* was bound (Fig. 2B, middle), these eight mutants strongly bound to both LEL and STL; however, the binding to WGA was not clear in Ω*cps1A*, Ω*cps1B*, and Ω*cps1G*. The other four lectins, rDiscoidin II, rOrysata, rBanana, and CSA, to which YIT 9029 (WT) bound (Fig. 2B, left), had different lectin-binding properties (26–32). Although binding to CSA was observed in these eight mutants, binding affinities to rDiscoidin II, rOrysata, and rBanana were different for each strain. Ω*cps1F*, Ω*cps1G*, and Ω*cps1J* strongly bound to these three lectins. Ω*cps1A*, Ω*cps1B*, and Δ*cps1C* weakly bound to those lectins. It was distinctive that Ω*cps1D* and Ω*cps1E* only bound to rBanana (29, 30).

Moreover, the lectin binding profile of Ω*cps1G* was significantly different from other *cps1* gene-disrupted strains. Ω*cps1G* strongly bound to not only extensive lectins such as rDiscoidin II, rOrysata, rBanana, CSA, LEL, and STL (26–36), but also to other lectins such as LFA, RCA120, rGal9N, BPL, rF17AG, rGRFT, CCA, Heltuba, rHeltuba, rCalsepa, rAOL, rGC2, ACA, and rMalectin. The sugar-binding specificities of lectins are listed in Table S4.

Among the genes essential for LCPS-1 biosynthesis in YIT 9029 (20), lectin-binding properties differed greatly depending on the gene. These results strongly suggest that the effect on the cell surface structure changes specifically and drastically for each gene. Based on these results, it was possible to analyze the similarity of gene functions by comparing the lectin-binding properties of genetically disrupted strains of YIT 9029.

### Profiling 51 mutants of YIT 9029

**Fig 5:**
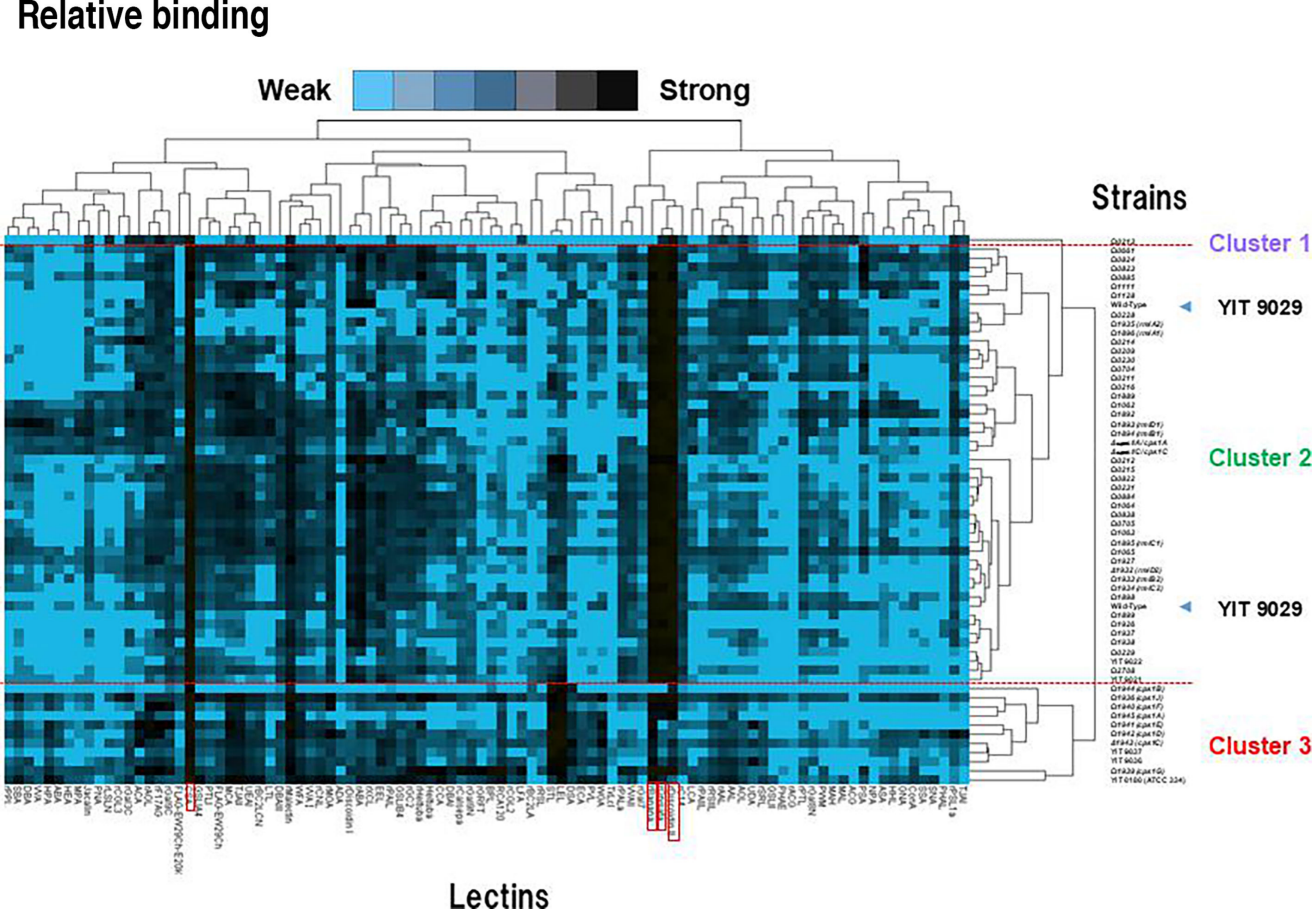
Relative binding of 51 mutants of *L. paracasei* strain Shirota (YIT 9029) with respect to lectin binding. Lectin-binding profiles of 51 mutants of YIT 9029 (WT) and WT with 96 lectin probes were compared using advanced lectin microarray technology (37). YIT 9021 (24), YIT 9022, YIT 9036, YIT 9037, and *L. casei* ATCC 334 were tested. The lectin-binding signals for each strain were normalized to the highest signal. The levels of lectin-binding signals are indicated by color change from blue (low binding levels) to black (high binding levels). The lectin binding properties of all tested strains were classified into three major clusters, from above, clusters one, two, and three. Cluster one classified Ω*0213* alone, and that did not bind to the mannose binder (rBanana) and weakly bound to rOrysata. Cluster two classified 43 mutants of YIT 9029 with YIT 9029 bound to an O-glycan binder (rDiscoidin II), two mannose binders (rOrysata, rBanana), and a Rha binder (CSA). Clusters one and two had mutants of all positive strains and slightly positive strains except for Ω*cps1F* to YIT 9029-specific MAb reactivity. YIT 9021 (24), YIT 9022, Δ*cps1A/cps1A*, and Δ*cps1C/cps1C* were also classified in cluster two. Cluster three included Ω*cps1A*, Ω*cps1B*, Δ*cps1C*, Ω*cps1D*, Ω*cps1E*, Ω*cps1F*, Ω*cps1G*, Ω*cps1J*, YIT 9036, and YIT 9037. All these mutants had negative and only one had slightly positive strain (Ω*cps1F*) to YIT 9029-specific MAb reactivity. Moreover, Δ*cps1A/cps1A* was a positive strain to YIT 9029-specific MAb reactivity (20).

Based on the above results, a statistical analysis was performed to compare the lectin-binding profiles of 51 mutants of YIT 9029 (WT), two complementary strains, and the WT with 96 lectin probes (Fig. 2A, Table S4) using advanced lectin microarray technology (37). Our laboratory collection strains with different antibody reactivity of YIT 9021 (24), YIT 9022, YIT 9036, and YIT 9037, and *L. casei* ATCC 334 were also tested. The lectin binding properties of all tested strains were classified into three major clusters: from the top, the clusters were named 1, 2, and 3 (Fig. 5). Clustering by lectin binding was consistent with the antibody reactivity (Table 2). Clusters 1 and 2 were composed of all positive strains and slightly positive strains to YIT 9029-specific Mab (21), except for Ω*cps1F*. Cluster 1 consisted of Ω*0213* alone, and it did not bind to the Man-binders (rBanana and rOrysata) (27–30). Cluster 2 included the largest number of members: 43 mutants and WT (YIT 9029). These mutant strains bound to an O-glycan binder (rDiscoidin II), two Man binders (rOrysata and rBanana), and a Rha binder (CSA) (26–32). YIT 9021 (24), YIT 9022, Δ*cps1A/cps1A*, and Δ*cps1C/cps1C* were also classified into cluster 2.

Cluster 3 included Ω*cps1A*, Ω*cps1B*, Δ*cps1C*, Ω*cps1D*, Ω*cps1E*, Ω*cps1F*, Ω*cps1G*, Ω*cps1J*, YIT 9036, and YIT 9037, all of which showed negative binding to YIT 9029-specific Mab (21), except for Ω*cps1F*, which was slightly positive to the antibody binding. Moreover, based on the lectin-binding affinities of our four laboratory collections with different antibodies, YIT 9021 (24) and YIT 9022, and YIT 9036 and YIT 9037 were categorized in cluster 2 and cluster 3, respectively.

It was confirmed that the lectin-binding properties of the gene-disrupted strains closely matched the reactivity of these mutants with YIT 9029-specific Mab (21) with some exceptions. For the present analysis, we included some gene knockouts within the same predicted operon because genes in the same operon (transcriptional unit) often produce one final product. For this, the following four possible operon genes were chosen: Ω*0209* to Ω*0216*, Ω*0228* to Ω*0231*, Ω*0822* to Ω*0824*, and Ω*1062* to Ω*1065*. The lectin-binding profiles of the mutants within the same operon showed certain similarities. Cluster 2, composed of 43 mutants, showed profiles similar to that of YIT 9029. Though all mutants of this cluster bound to Man-binders (rOrysata and rBanana), only Ω*0213* cells did not bind to rBanana (18). For this reason, only Ω*0213* cells were classified as a different branch from other strains, which were classified under cluster 1. The reasons why only Ω*0213* was clustered in cluster 1, and all mutants of YIT 9029 bound to the Rha binder CSA (34) are discussed in the Discussion.

## DISCUSSION

It is well documented that LAB used in food are beneficial for human health; however, the interaction between bacterial cell surface components and host immune cells is limited (38). We investigated *Lacticaseibacillus paracasei* strain Shirota (YIT 9029) for its immunological action against host cells, and it plays a critical role in the modulation of immune cells (20). YIT 9029 is a cured strain of the bacteriophage FSW of *Lactobacillus casei* YIT 9018 (39), and both strains react with YIT 9029-specific Mab (21). It has unique PSs called LCPS-1 (18) and LCPS-2 (19) on its cell surface, and LCPS-1 greatly affects the physiological effects of this bacterium (20).

Based on the previous reports (20, 37), we investigated how each of the genes involved in cell surface PS synthesis of YIT 9029 affects the actual structure of this bacterium by analyzing which genes are linked to which lectin-binding properties. To answer these questions, we predicted 56 genes involved in cell wall polysaccharide biosynthesis of YIT 9029 from reported bacterial glycosyltransferases, etc., and 51 genes were disrupted (Fig. 1, Table 1). As a technical challenge, lectin microarray analysis was initially developed to analyze mammalian cells. However, it was successfully applied to analyze YIT 9029 mutants using an advanced version of the 96 lectin microarray (25) (Fig. 2A, Table S4). Hence, a series of gene disruption mutants of glycosylation genes and modification enzymes of YIT 9029 were constructed, and the structure and functional relationships of the cell surface molecules of YIT 9029 were elucidated in more detail. Furthermore, we compared their binding profiles to lectins in parallel with their binding abilities to YIT 9029-specific Mab, which is our original tool (21).

We showed that the Rha-binding lectin CSA (formerly CSL) binds to YIT 9029 (37). Three new lectins were identified that bind to YIT 9029; those were an O-glycan binder (rDiscoidin II) and two Man-binders (rOrysata/rBanana) (Fig. 2B, left). The binding affinities between YIT 9029 and Man-binding lectins, rOrysata/rBanana, were inhibited by 1 mM Man (Fig. 3). It is suggested that these two lectins bind to Man of the cell surface molecules in YIT 9029, but no Man was found in the cell wall PSs of YIT 9029 (18, 19). Although these facts may seem contradictory at first glance, based on the research of Sharon et al. (40), lectins typically contain two or more carbohydrate-combining sites per molecule. Generally, galactose-specific lectins do not react with glucose, and glucose-specific lectins do not react with galactose (40). We consider that the OH group at the fourth position of the Man is particularly important in the binding between rOrysata/rBanana and YIT 9029. Therefore, even if the presence of Man is not detected in YIT 9029, it is not inconsistent for the Man-binding lectin to bind to it.

In this study, several remarkable points were observed through the lectin microarray analyses. (i) The loss of LCPS-1 did not influence the number of lectins capable of binding to mutant cells. We considered LCPS-1 as a suppressive molecule that induces cytokine production by macrophages owing to the physical masking effect of LCPS-1 on the cell surface (20). (ii) The Ω*1939* (*cps1G*) and Ω*1936* (*cps1J*) mutants still have the affinity to rDiscoidin II, rOrysata, and rBanana, which are possible markers for LCPS-1 biosynthesis, although these mutants do not react with YIT 9029-specific MAb reactivity, nor produce LCPS-1 (20). (iii) The similarity of the lectin-binding profiles of Ω*1942* (*cps1D*) and Ω*1941* (*cps1E*) mutants probably indicates that these gene products have a similar role in the synthesis and maturation of LCPS-1. (iv) The Ω*1939* (*cps1G*) mutant dramatically changes the binding pattern of lectins, indicating big changes in the surface structure of the mutant cells. All these indications and assumptions will be addressed in future research.

The mutation that reduced antibody reactivity in YIT 9029 was confirmed to be a deletion of LCPS-1 (20). However, it is unclear whether the effects of LCPS-1 deletion on the cell surface PS structures of YIT 9029 cells are uniform or diverse. Here, we demonstrated that the mutations in the cell surface PS that occur after the deletion of LCPS-1 vary greatly depending on the gene.

Another interesting result is that while Ω*0213* develops LCPS-1 on the cell wall, the lectin binding profile is different from that of YIT 9029 (Fig. 5). In this mutant, the *CDS0213* gene encoding a putative PS biosynthesis protein (CAQ65366.1) (23) was knocked out, and the sequence of *CDS0213* was similar to eps7I of *L. casei* strain BL23 (23) (Table 1). This gene is widely conserved in bacterial genomes and is classified as the Glycosyltransferase Family 32 (http://www.cazy.org/GT32.html) with known activity as α-1,6-mannosyltransferase (EC 2.4.1.232). This result was a good match between the predicted gene function and lectin-binding in the bacteria.

On the other hand, among the 51 genes chosen for disruption, several groups of genes may form operons, including Ω*0209* to Ω*0216*, Ω*0228* to Ω*0231*, Ω*0822* to Ω*0824*, and Ω*1062* to Ω*1065*. As shown in Fig. 5, we confirmed that the lectin-binding profiles of the mutants within the same operon have certain similarities.

These observations suggest that the combined use of lectin microarray technology and a mutant strain library of YIT 9029 is a powerful tool for identifying unknown bacterial gene functions in terms of cell surface glycome, which is a novel approach to glycophenotyping. It is important to clarify the relationship between the phenotype and genotype of bacterial strains. The use of lectins in combination is an effective solution when antibodies cannot be produced by the bacterial strain.

**Fig 6:**
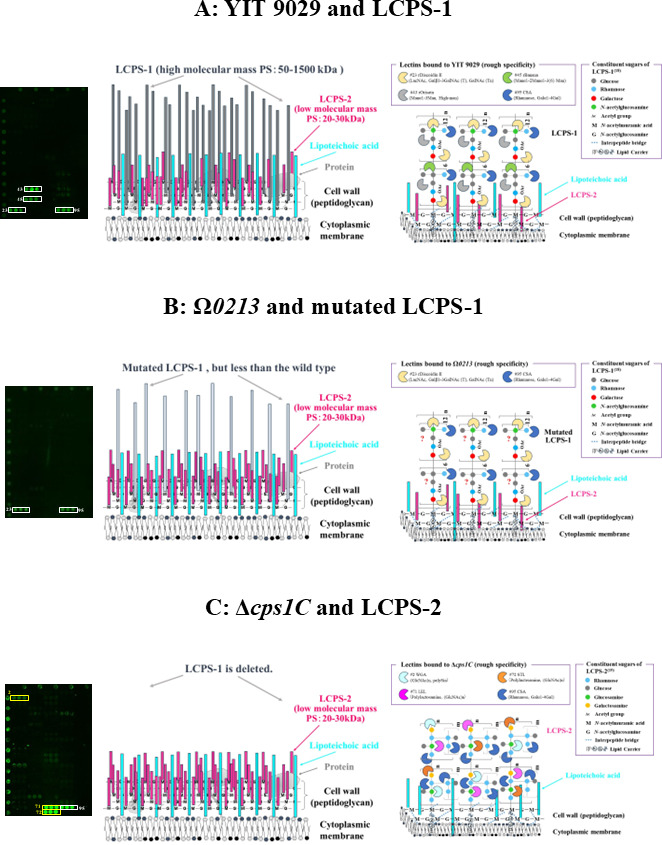
Possible lectin-binding profiles of wild-type YIT 9029 (A) and its mutant strains Ω*0213* (B) and Δ*cps1C* (C). From left to right, each panel shows lectin-binding affinities, a schematic illustration of cell-surface PS structures, and lectins bound to cell-surface PS molecules. (A) YIT 9029 has high and low molecular mass PSs named LCPS-1 (18) and LCPS-2 (19). (B) Ω*0213* is predicted to have fewer LCPS-1 molecules per cell than YIT 9029, or it has modified LCPS-1 molecules. (C) Δ*cps1C* is completely missing LCPS-1 (20). Here, the lectin-binding profiles of YIT 9029 and Δ*cps1C* are duplicated for comparison with Ω*0213*.

We summarized the relationship among the lectin binding profile, the predicted cell surface structures, and the lectins bound to the cell surface PS molecules to focus on wild-type YIT 9029 (cluster 2), its mutant strains Ω*0213* (cluster 1), and Δ*cps1C* (cluster 3) (Fig. 6). From left to right, each panel shows lectin-binding affinities, schematic illustration of cell-surface PS structures, and lectins bound to cell-surface PS molecules. Here, the lectin-binding profiles of YIT 9029 and Δ*cps1C* are duplicated for comparison with Ω*0213*.

Panel A: YIT 9029, classified into cluster 2, has high and low molecular mass PSs named LCPS-1 (18) and LCPS-2 (19). Considering the structure of LCPS-1 (18), CSA and rDiscoidin II are predicted to bind to Rha, the acetyl group, and lactose of LCPS-1. Though LCPS-1 has Glc at the non-reducing end, none of the lectins used in this study (Fig. 2A, Table S4) recognize glucose located at the non-reducing terminal. Therefore, Man-binding lectins (rOrisata/rBanana) are predicted to bind to two Glc molecules of LCPS-1 (26–32).

Panel B: Ω*0213,* classified into cluster 1, is predicted to have fewer LCPS-1 molecules per cell than those of YIT 9029, or it has modified LCPS-1 molecules from the results of the ability of the mutants to bind to YIT 9029-specific MAb (Table 2; Table S3). We predict rDiscoidin II and CSA bind to mutated LCPS-1. To analyze the structure of LCPS-1 in this strain is a challenge for the future.

Panel C: Δ*cps1C,* classified into cluster 3, is completely missing LCPS-1 (20), and we predict WGA, LEL, STL, and CSA bind to LCPS-2 (19). Considering the structure of LCPS-2 (19), CSA is predicted to recognize Rha of LCPS-2 (37), and WGA, LEL, and STL are predicted to recognize the acetyl group in glucosamine and galactosamine of LCPS-2 (33–36). The existence of LTA has been suggested in YIT 9029 (41), but its structure has not been elucidated. Regarding the lectins that bind to LTA, this is a point to focus on in the future. In conclusion, the combined use of lectin microarray technology and a mutant strain library of YIT 9029 makes a powerful tool for identifying unknown bacterial gene functions in terms of cell surface glycome. This is a novel approach to glycophenotyping, and lectin microarrays enable the identification of the cell wall glycome in relation to bacterial gene function.

## MATERIALS AND METHODS

### Criteria for the selection of genes from YIT 9029 and annotation of the genes

The genes predicted to be involved in the biosynthesis of cell wall-associated PSs of YIT 9029 (AB470649, LCS853105–LCS853145, and GRN 429) were selected from the in-house genome laboratories’ data (T. Sato, et al., unpublished data; Table 1, left). These were based on the similarity of at least 20% of the amino acid sequences with other bacterial proteins known or predicted to be involved in extracellular or cell wall PSs biosynthesis in the GenBank database. In addition, genes were selected to contain motifs for glycosyl transferases and glycosylation enzymes in the genome of *L. casei* BL23 (23). The protein ID is listed in Table 1 (rightmost).

### Bacterial strains and plasmids used in this study

The bacterial strains and plasmids used in this study are listed in Table S1. Fifty-one gene knockout mutants of YIT 9029 were employed for the analysis; some were newly constructed as described below, while others were from a previous study (20). *L. casei* ATCC 334 (YIT 0180), a neotype strain of *L. casei* (42), was purchased from the American Type Culture Collection (Manassas, VA). We tested four strains from our laboratory collection with different antibody reactivities: YIT 9021 (24), YIT 9022, YIT 9036, and YIT 9037. *Escherichia coli* JM109 was purchased from Toyobo Co., Ltd. (Osaka, Japan) as competent cells for DNA transformation.

### Gene manipulation of YIT 9029 based on the homologous recombination principle

Forty-nine mutants of YIT 9029 designated “Ω” were produced by insertion of a plasmid with the respective truncated gene fragment deleting both N- and C-terminal coding regions as described previously (Table S1). The synthetic primers used to amplify the truncated gene fragments are listed in Table S2. All mutants produced by one-step homologous recombination were cultured in De Man–Rogosa–Sharpe (MRS) medium (Becton, Dickinson and Company, New Jersey, USA) containing 10 µg/mL erythromycin (39, 43) under static culture conditions. The mutant Δ*1932* (*rmlD2*) of YIT 9029 was constructed by two-step homologous recombination and described as Δ*cps1C* (20). The synthetic primer set for amplification of N- and C-terminal fragments is shown in Table S2. The plasmid pRD8 to isolate the mutant Δ*1932* (*rmlD2* deficient) of YIT 9029 is described in Fig. S1.

### Reagents and chemicals for recombinant DNA technology

The reagents and DNA technology have been described in a previous report (20). Briefly, DNA was amplified by polymerase chain reaction (PCR) using KOD PLUS DNA polymerase (TOYOBO Co., Ltd., Osaka, Japan) or TaKaRa Ex Taq (Takara Bio Inc., Otsu, Japan). Restriction endonucleases, calf intestinal alkaline phosphatase, and a DNA Ligation Kit were purchased from Takara Bio Inc. or TOYOBO Co., Ltd. Plasmid purification was performed using the Wizard Plus SV Minipreps DNA Purification System (Promega K.K., Tokyo, Japan), and DNA fragments amplified by PCR were purified using the Qiaquick Gel Extraction Kit (QIAGEN K.K., Tokyo, Japan). Custom-made synthetic DNAs were purchased from Sigma-Aldrich Japan K.K. (Tokyo, Japan).

### Recombinant plasmid construction and insertion, and deletion mutagenesis

The plasmids used in this study are listed in Table S1. The basic procedures for constructing recombinant plasmids for insertional mutagenesis, deletion mutagenesis, and isolation of bacterial clones harboring these chromosomal mutations have been described previously (20). Plasmid pRD8 was constructed as follows: DNA fragments containing N- and C-terminal coding regions of the *CDS1932* (*rmlD2*) gene were amplified by PCR using the primers shown in Table S2, and the purified DNA fragments thus obtained were digested with the respective restriction enzymes. These two fragments and pBE31 (43), digested with *Kpn* I and *Xba* I, were mixed and ligated to obtain an in-frame deletion fragment of the *rmlD2* gene, which was cloned on pBE31 (43). The resulting plasmid was named pRD8 (Fig. S1). Construction of deletion mutants of YIT 9029 at *cps1A* (Δ*cps1A*) and *cps1C* (Δ*cps1C*), and each revertant harboring the respective wild-type (WT) gene in *trans* on the chromosome (Δ*cps1A,* Δ*cps1A/cps1A*, and *Δcps1C/cps1C*) was described previously (20). All primers for deletion mutagenesis were designed to enable in-frame rejoining of the N- and C-terminal peptide fragments of the gene, thereby avoiding translational interruptions within an operon. YIT 9029 was transformed with these plasmids, and erythromycin-resistant clones (39, 42) were selected. These clones contained recombinant plasmids integrated into either side of the respective gene fragments via homologous recombination. After several cycles of subculturing (one thousandth inoculation into fresh medium, followed by full growth), erythromycin-sensitive clones were screened and checked for reversion or deletion.

### Culture of bacterial strains and storage

Bacterial cells were cultured in 4 mL of MRS medium with or without erythromycin (10 µg/mL) for 22–24 h at 37°C under static culture conditions. After culturing (total cells: 1 × 10^9^–2 × 10^10^), the turbidity of the cultures was measured using a Klett-Summerson spectrophotometer (Klett MFG, New York, USA). Because the Klett value increased linearly with culture time up to 23 h (Fig. S2), the culture time for each mutant strain was fixed to be 22 h in this study. Then the cells were labeled with 10 µM SYTOX Orange Nucleic Acid Stain (44) (Molecular Probes Co., Ltd.) in phosphate-buffered saline (PBS) containing 1% bovine serum albumin (BSA) for the lectin microarray analysis. The fluorescence intensity of 2 × 10^8^ labeled cells was measured using an ARVO X3 apparatus (PerkinElmer) after 1 h of labeling. The fluorescence of cells labeled with SYTOX Orange (44) was stabilized by freezing at −20°C for 2 weeks. Hence, we stored labeled cells at −20°C and used them within 2 weeks. Fluorescence intensities for all the tested strains were adjusted to within two times (Fig. S3). Before testing for microarray, the cells were suspended in 360 µL of PBS containing 1% BSA (PBS/BSA) (37).

### Determination of the reactivity of mutants to YIT 9029-specific monoclonal antibody

The reactivity of the mutants to YIT 9029-specific MAb (21) was determined using a sandwich ELISA, as described previously (20). The resultant fluorescence intensities of WT (YIT 9029) and mutants were classified into three types: “positive” with full to half of the color intensity that WT showed, “slightly positive” with weak or slight color intensity, and “negative” with very weak or no color. An absorbance of ≥1.0 and <0.25 was considered positive and negative. The absorbance of ≥0.25 but <1.0 was considered slightly positive.

### Lectin microarray hybridization

The lectin microarray was prepared as described previously (25, 37, 45). Briefly, 96 lectins were dissolved at a concentration of 0.5 mg/mL in a spotting solution (Matsunami Glass) and spotted onto epoxysilane-coated glass slides (Schott) in triplicate using a non-contact microarray-printing robot (MicroSys4000; Genomic Solutions, Ann Arbor, MI). The origins and binding specificities of the tested lectins are listed in Table S4. YIT 9029, mutants of YIT 9029, and other tested strains were labeled with SYTOX Orange (44) as described above, then were added to each well of a glass slide containing immobilized lectins (1–2 × 10^9^ cells/100 µL/well) followed by incubation at 4°C for 1 h. In this study, washing buffer at room temperature (RT) was used because no difference was observed in the binding of lectin to bacteria for the temperature of the washing buffer between RT and 4°C. Unbound cells were mildly removed by immersing the inverted lectin microarray glass slides in more than 1 L of PBS at RT for 30 min. Cells bound with lectins immobilized on a glass slide were detected using an evanescent-field fluorescence scanner. Data are shown as the ratio of the fluorescence intensities of the 96 lectins (31) relative to the maximal fluorescence intensity on the lectin microarray. Levels of lectin-binding signals are indicated by a color change from blue (low binding levels) to black (high binding levels).

### Effects of simple saccharides addition on the lectin microarray assay

To determine the effects of mono- and di-saccharides addition on the binding affinity of YIT 9029 to four lectins, rDiscoidin II, rBanan, Orysata, and CSA, the following saccharides were added to the assay system at a concentration of 1 or 50 mM: D-galactopyranose (Gal), D-glucopyranose (Glc), D-galactosylpyranosyl-(β1→4)-D-Glc (Lac), D-mannopyranose (Man), L-rhamnopyranose (Rha), D-fructofuranosyl-(2↔1)-D-glucopyranoside (Suc), or (3S,4R,5S,6S)-6-methyltetrahydro-2H-pyran-2,3,4,5-tetraol (Fuc). The efficiency of the inhibition of YIT 9029 binding to these lectins is shown as the ratio of the fluorescence intensity with each saccharide to that of the control with no added saccharides (36).

### Profiling 51 mutants of YIT 9029

We compared the lectin-binding profiles of 51 YIT 9029 mutants (Table 1), YIT 9029 (WT), YIT 9022, YIT 9036, YIT 9037, and *L. casei* ATCC 334 (YIT 0180) with 96 lectin probes (Fig. 2A, Table S4) using advanced lectin microarray technology (36). To evaluate the similarity of gene functions, cluster analysis was performed to relate the cell wall PS biosynthesis genes in YIT 9029 and the lectin binding profiles. Considering the error between the arrays, YIT 9029 used two arrays in the comparison between the arrays. Unsupervised clustering was performed by employing the average linkage method using open-source Cluster 3.0 software developed by Michael Eisen of the Berkeley Lab. A heat map with clustering was generated using Java Treeview (Fig. 5).

## SUPPLEMENTAL MATERIAL

Figure S1 aem.01707-24-s0001.tif Preparation of plasmid pRD8.

Figure S2 aem.01707-24-s0002.tif Klett units.

Figure S3 aem.01707-24-s0003.tif Fluorescence intensity of all tested strains.

Supplemental legends aem.01707-24-s0004.docx Legends for Fig. S1 to S3.

Table S1 aem.01707-24-s0005.docx Bacterial strains used in this study.

Table S2 aem.01707-24-s0006.docx Synthetic primers for truncated amplification.

Table S3 aem.01707-24-s0007.docx Reactivity of *L. paracasei* Shirota (YIT 9029) and mutants to *L. paracasei* Shirota-specific monoclonal antibody (MAb).

Table S4 aem.01707-24-s0008.docx Sugar-binding specificities of the lectins in this study.

ASM does not own the copyrights to Supplemental Material that may be linked to, or accessed through, an article. The authors have granted ASM a non-exclusive, world-wide license to publish the Supplemental Material files. Please contact the corresponding author directly for reuse.

## References

[1] Chapot-Chartier MP, Kulakauskas S. 2014. Cell wall structure and function in lactic acid bacteria. Microb Cell Fact 13 Suppl 1:S9. doi:10.1186/1475-2859-13-S1-S9 25186919 PMC4155827

[2] Weintraub A. 2003. Immunology of bacterial polysaccharide antigens. Carbohydr Res 338:2539–2547. doi:10.1016/j.carres.2003.07.008 14670715

[3] Mazmanian SK, Kasper DL. 2006. The love-hate relationship between bacterial polysaccharides and the host immune system. Nat Rev Immunol 6:849–858. doi:10.1038/nri1956 17024229

[4] Campanero-Rhodes MA, Palma AS, Menéndez M, Solís D. 2019. Microarray strategies for exploring bacterial surface glycans and their interactions with glycan-binding proteins. Front Microbiol 10:2909. doi:10.3389/fmicb.2019.02909 32010066 PMC6972965

[5] Vinogradov E, Sadovskaya I, Grard T, Chapot-Chartier MP. 2016. Structural studies of the rhamnose-rich cell wall polysaccharide of Lactobacillus casei BL23. Carbohydr Res 435:156–161. doi:10.1016/j.carres.2016.10.002 27756016

[6] Zeidan AA, Poulsen VK, Janzen T, Buldo P, Derkx PMF, Øregaard G, Neves AR. 2017. Polysaccharide production by lactic acid bacteria: from genes to industrial applications. FEMS Microbiol Rev 41:S168–S200. doi:10.1093/femsre/fux017 28830087

[7] Shida K, Nanno M, Nagata S. 2011. Flexible cytokine production by macrophages and T cells in response to probiotic bacteria: a possible mechanism by which probiotics exert multifunctional immune regulatory activities. Gut Microbes 2:109–114. doi:10.4161/gmic.2.2.15661 21637028

[8] Shida K, Nomoto K. 2013. Probiotics as efficient immunopotentiators: translational role in cancer prevention. Indian J Med Res138:808–814. PMC3928711 24434333

[9] Matsumoto K, Takada T, Shimizu K, Kado Y, Kawakami K, Makino I, Yamaoka Y, Hirano K, Nishimura A, Kajimoto O, Nomoto K. 2006. The effects of a probiotic milk product containing Lactobacillus casei strain Shirota on the defecation frequency and the intestinal microflora of sub-optimal health state volunteers: a randomized placebo-controlled cross-over study. Bioscience Microflora 25:39–48. doi:10.12938/bifidus.25.39

[10] Takada M, Nishida K, Kataoka-Kato A, Gondo Y, Ishikawa H, Suda K, Kawai M, Hoshi R, Watanabe O, Igarashi T, Kuwano Y, Miyazaki K, Rokutan K. 2016. Probiotic Lactobacillus casei strain Shirota relieves stress-associated symptoms by modulating the gut-brain interaction in human and animal models. Neurogastroenterol Motil 28:1027–1036. doi:10.1111/nmo.12804 26896291

[11] Kato-Kataoka A, Nishida K, Takada M, Kawai M, Kikuchi-Hayakawa H, Suda K, Ishikawa H, Gondo Y, Shimizu K, Matsuki T, Kushiro A, Hoshi R, Watanabe O, Igarashi T, Miyazaki K, Kuwano Y, Rokutan K. 2016. Fermented milk containing Lactobacillus casei strain Shirota preserves the diversity of the gut microbiota and relieves abdominal dysfunction in healthy medical students exposed to academic stress. Appl Environ Microbiol 82:3649–3658. doi:10.1128/AEM.04134-15 27208120 PMC4959178

[12] Takada M, Nishida K, Gondo Y, Kikuchi-Hayakawa H, Ishikawa H, Suda K, Kawai M, Hoshi R, Kuwano Y, Miyazaki K, Rokutan K. 2017. Beneficial effects of Lactobacillus casei strain Shirota on academic stress-induced sleep disturbance in healthy adults: a double-blind, randomised, placebo-controlled trial. Benef Microbes 8:153–162. doi:10.3920/BM2016.0150 28443383

[13] Cox AJ, Makino H, Cripps AW, West NP. 2019. Recovery of Lactobacillus casei strain Shirota (LcS) from faeces with 14 days of fermented milk supplementation in healthy Australian adults. Asia Pac J Clin Nutr 28:734–739. doi:10.6133/apjcn.201912_28(4).0009 31826370

[14] Otaka M, Kikuchi-Hayakawa H, Ogura J, Ishikawa H, Yomogida Y, Ota M, Hidese S, Ishida I, Aida M, Matsuda K, Kawai M, Yoshida S, Kunugi H. 2021. Effect of Lacticaseibacillus paracasei strain Shirota on improvement in depressive symptoms, and its association with abundance of actinobacteria in gut microbiota. Microorganisms 9:1026. doi:10.3390/microorganisms9051026 34068832 PMC8150707

[15] Cook CM, Makino H, Kato K, Blonquist T, Derrig L, Shibata H. 2023. The probiotic Lacticaseibacillus paracasei strain Shirota (LcS) in a fermented milk beverage survives the gastrointestinal tract of generally healthy U.S. adults. Int J Food Sci Nutr 74:645–653. doi:10.1080/09637486.2023.2246693 37584253

[16] Kikuchi-Hayakawa H, Ishikawa H, Suda K, Gondo Y, Hirasawa G, Nakamura H, Takada M, Kawai M, Matsuda K. 2023. The probiotic Lacticaseibacillus paracasei strain Shirota (LcS) in a fermented milk beverage survives the gastrointestinal tract of generally healthy U.S. adults. Nutrients 15:5119. doi:10.3390/nu15245119 38140378 PMC10745872

[17] Takeda K, Okumura K. 2007. Effects of a fermented milk drink containing Lactobacillus casei strain Shirota on the human NK-cell activity. J Nutr 137:791S–3S. doi:10.1093/jn/137.3.791S 17311976

[18] Mizukoshi H, Kimura K, Ikemura H, Mori Y, Nagaoka M. 2022. Structural determination of the cell wall polysaccharide LCPS-1 in Lacticaseibacillus paracasei strain Shirota YIT 9029. Carbohydr Res 521:108670. doi:10.1016/j.carres.2022.108670 36103733

[19] Nagaoka M, Muto M, Nomoto K, Matuzaki T, Watanabe T, Yokokura T. 1990. Structure of polysaccharide-peptidoglycan complex from the cell wall of Lactobacillus casei YIT9018. J Biochem 108:568–571. doi:10.1093/oxfordjournals.jbchem.a123243 2292584

[20] Yasuda E, Serata M, Sako T. 2008. Suppressive effect on activation of macrophages by Lactobacillus casei strain shirota genes determining the synthesis of cell wall-associated polysaccharides . Appl Environ Microbiol 74:4746–4755. doi:10.1128/AEM.00412-08 18552190 PMC2519339

[21] Yuki N, Watanabe K, Mike A, Tagami Y, Tanaka R, Ohwaki M, Morotomi M. 1999. Survival of a probiotic, Lactobacillus casei strain Shirota, in the gastrointestinal tract: selective isolation from faeces and identification using monoclonal antibodies. Int J Food Microbiol 48:51–57. doi:10.1016/s0168-1605(99)00029-x 10375134

[22] Graninger M, Kneidinger B, Bruno K, Scheberl A, Messner P. 2002. Homologs of the Rml enzymes from Salmonella enterica are responsible for dTDP-β-L-rhamnose biosynthesis in the gram-positive thermophile Aneurinibacillus thermoaerophilus DSM 10155. Appl Environ Microbiol 68:3708–3715. doi:10.1128/AEM.68.8.3708-3715.2002 12147463 PMC124034

[23] Mazé A, Boël G, Zúñiga M, Bourand A, Loux V, Yebra MJ, Monedero V, Correia K, Jacques N, Beaufils S, Poncet S, Joyet P, Milohanic E, Casarégola S, Auffray Y, Pérez-Martínez G, Gibrat JF, Zagorec M, Francke C, Hartke A, Deutscher J. 2010. Complete genome sequence of the probiotic Lactobacillus casei strain BL23. J Bacteriol 192:2647–2648. doi:10.1128/JB.00076-10 20348264 PMC2863562

[24] Watanabe K, Ishibashi K, Nakashima Y, Sakurai T. 1984. A phage-resistant mutant of Lactobacillus casei which permits phage adsorption but not genome injection. J Gen Virol 65 (Pt 5):981–986. doi:10.1099/0022-1317-65-5-981 6427405

[25] Tateno H, Toyota M, Saito S, Onuma Y, Ito Y, Hiemori K, Fukumura M, Matsushima A, Nakanishi M, Ohnuma K, Akutsu H, Umezawa A, Horimoto K, Hirabayashi J, Asashima M. 2011. Glycome diagnosis of human induced pluripotent stem cells using lectin microarray. J Biol Chem 286:20345–20353. doi:10.1074/jbc.M111.231274 21471226 PMC3121447

[26] Barondes SH, Cooper DN, Haywood-Reid PL. 1983. Discoidin I and discoidin II are localized differently in developing dictyostelium discoideum. J Cell Biol 96:291–296. doi:10.1083/jcb.96.1.291 6826651 PMC2112257

[27] Peumans WJ, Barre A, Houles Astoul C, Rovira P, Rougé P, Proost P, Truffa-Bachi P, Jalali AAH, Van Damme EJM, Zhang W. 2000. Isolation and characterization of a jacalin-related mannose-binding lectin from salt-stressed rice (Oryza sativa) plants. Planta 210:970–978. doi:10.1007/s004250050705 10872230

[28] Nagae M, Mishra SK, Hanashima S, Tateno H, Yamaguchi Y. 2017. Distinct roles for each N-glycan branch interacting with mannose-binding type Jacalin-related lectins Orysata and Calsepa. Glycobiology 27:1120–1133. doi:10.1093/glycob/cwx081 28973127

[29] Mo H, Winter HC, Van Damme EJ, Peumans WJ, Misaki A, Goldstein IJ. 2001. Carbohydrate binding properties of banana (Musa acuminata) lectin I. Novel recognition of internal alpha1,3-linked glucosyl residues. Eur J Biochem 268:2609–2615. doi:10.1046/j.1432-1327.2001.02148.x 11322880

[30] Singh SS, Devi SK, Ng TB. 2014. Banana lectin: a brief review. Molecules 19:18817–18827. doi:10.3390/molecules191118817 25407720 PMC6272006

[31] Mistou M-Y, Sutcliffe IC, van Sorge NM. 2016. Bacterial glycobiology: rhamnose-containing cell wall polysaccharides in Gram-positive bacteria. FEMS Microbiol Rev 40:464–479. doi:10.1093/femsre/fuw006 26975195 PMC4931226

[32] Shirai T, Watanabe Y, Lee M, Ogawa T, Muramoto K. 2009. Structure of rhamnose-binding lectin CSL3: unique pseudo-tetrameric architecture of a pattern recognition protein. J Mol Biol 391:390–403. doi:10.1016/j.jmb.2009.06.027 19524596

[33] Parasuraman P, Murugan V, Selvin JFA, Gromiha MM, Fukui K, Veluraja K. 2014. Insights into the binding specificity of wild type and mutated wheat germ agglutinin towards Neu5Acα(2-3)Gal: a study by in silico mutations and molecular dynamics simulations. J Mol Recognit 27:482–492. doi:10.1002/jmr.2369 24984865

[34] Itakura Y, Nakamura-Tsuruta S, Kominami J, Tateno H, Hirabayashi J. 2017. Sugar-binding profiles of chitin-binding lectins from the hevein family: a comprehensive study. Int J Mol Sci 18:1160. doi:10.3390/ijms18061160 28556796 PMC5485984

[35] Schallus T, Jaeckh C, Fehér K, Palma AS, Liu Y, Simpson JC, Mackeen M, Stier G, Gibson TJ, Feizi T, Pieler T, Muhle-Goll C. 2008. Malectin: a novel carbohydrate-binding protein of the endoplasmic reticulum and a candidate player in the early steps of protein N-glycosylation. Mol Biol Cell 19:3404–3414. doi:10.1091/mbc.e08-04-0354 18524852 PMC2488313

[36] Naito Y, Minamihara T, Ando A, Marutani T, Oguri S, Nagata Y. 2001. Domain construction of cherry-tomato lectin: relation to newly found 42-kDa protein. Biosci Biotechnol Biochem 65:86–93. doi:10.1271/bbb.65.86 11272850

[37] Yasuda E, Tateno H, Hirabayashi J, Iino T, Sako T. 2011. Lectin microarray reveals binding profiles of Lactobacillus casei strains in a comprehensive analysis of bacterial cell wall polysaccharides. Appl Environ Microbiol 77:4539–4546. doi:10.1128/AEM.00240-11 21602390 PMC3127709

[38] Abdul Hakim BN, Xuan NJ, Oslan SNH. 2023. A comprehensive review of bioactive compounds from lactic acid bacteria: potential functions as functional food in dietetics and the food industry. Foods 12:2850. doi:10.3390/foods12152850 37569118 PMC10417365

[39] Shimizu-Kadota M, Kiwaki M, Sawaki S, Shirasawa Y, Shibahara-Sone H, Sako T. 2000. Insertion of bacteriophage phiFSW into the chromosome of Lactobacillus casei strain Shirota (S-1): characterization of the attachment sites and the integrase gene. Gene 249:127–134. doi:10.1016/s0378-1119(00)00154-2 10831846

[40] Sharon N. 2009. Lectins, p 1–11. In Encyclopedia of life science. John Wiley & Sons, Ltd.

[41] Setoyama T, Nomoto K, Yokokura T, Mutai M. 1985. Protective effect of lipoteichoic acid from Lactobacillus casei and Lactobacillus fermentum against Pseudomonas aeruginosa in mice. Microbiology (Reading, Engl) 131:2501–2503. doi:10.1099/00221287-131-9-2501 3934337

[42] Dicks LM, Du Plessis EM, Dellaglio F, Lauer E. 1996. Reclassification of Lactobacillus casei subsp. casei ATCC 393 and Lactobacillus rhamnosus ATCC 15820 as Lactobacillus zeae nom. rev., designation of ATCC 334 as the neotype of L. casei subsp. casei, and rejection of the name Lactobacillus paracasei. Int J Syst Bacteriol 46:337–340. doi:10.1099/00207713-46-1-337 8573516

[43] Kiwaki M, Shimizu-kadota M. 2002. Development of genetic manipulation systems and the appli-cation to genetic research in Lactobacillus casei strain Shirota. Bioscience Microflora 20:121–129. doi:10.12938/bifidus1996.20.121

[44] Yan X, Habbersett RC, Cordek JM, Nolan JP, Yoshida TM, Jett JH, Marrone BL. 2000. Development of a mechanism-based, DNA staining protocol using SYTOX orange nucleic acid stain and DNA fragment sizing flow cytometry. Anal Biochem 286:138–148. doi:10.1006/abio.2000.4789 11038284

[45] Hirabayashi J, Yamada M, Kuno A, Tateno H. 2013. Lectin microarrays: concept, principle and applications. Chem Soc Rev 42:4443–4458. doi:10.1039/c3cs35419a 23443201

