# Supplementary figures and images for: Glycophenotyping of mutants of Lacticaseibacillus paracasei by lectin microarray
Source: Appl Environ Microbiol. 2025 Jul 9;91(8):e01707-24. doi: 10.1128/aem.01707-24 (PMC12366308; doi:10.1128/aem.01707-24)

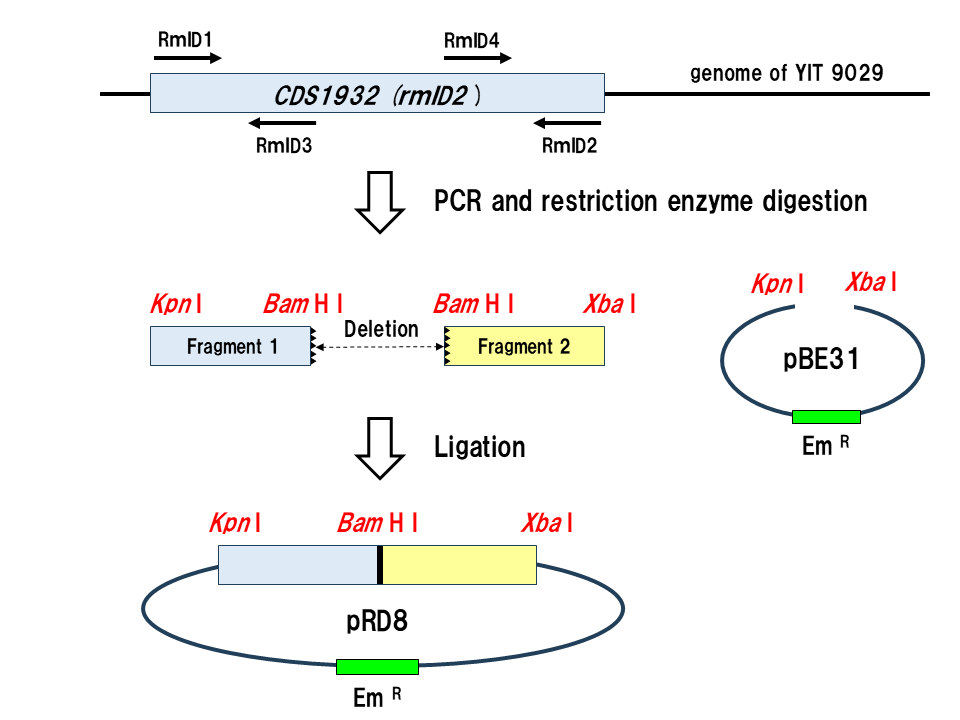

Supplement: Figure S1 — Preparation of plasmid pRD8. [file aem.01707-24-s0001.tif]

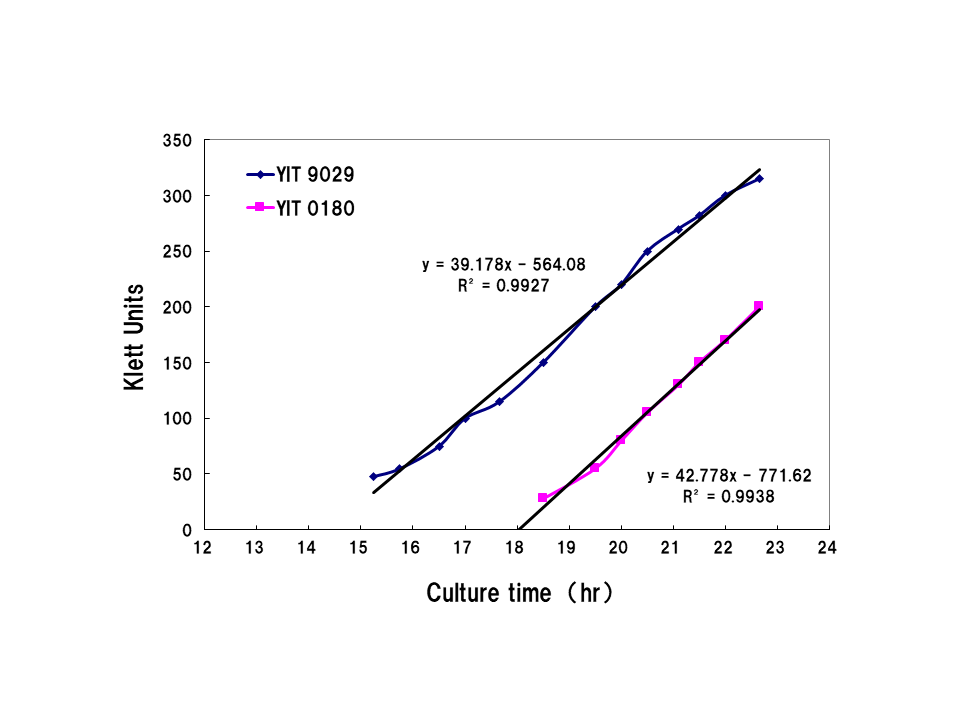

Supplement: Figure S2 — Klett units. [file aem.01707-24-s0002.tif]

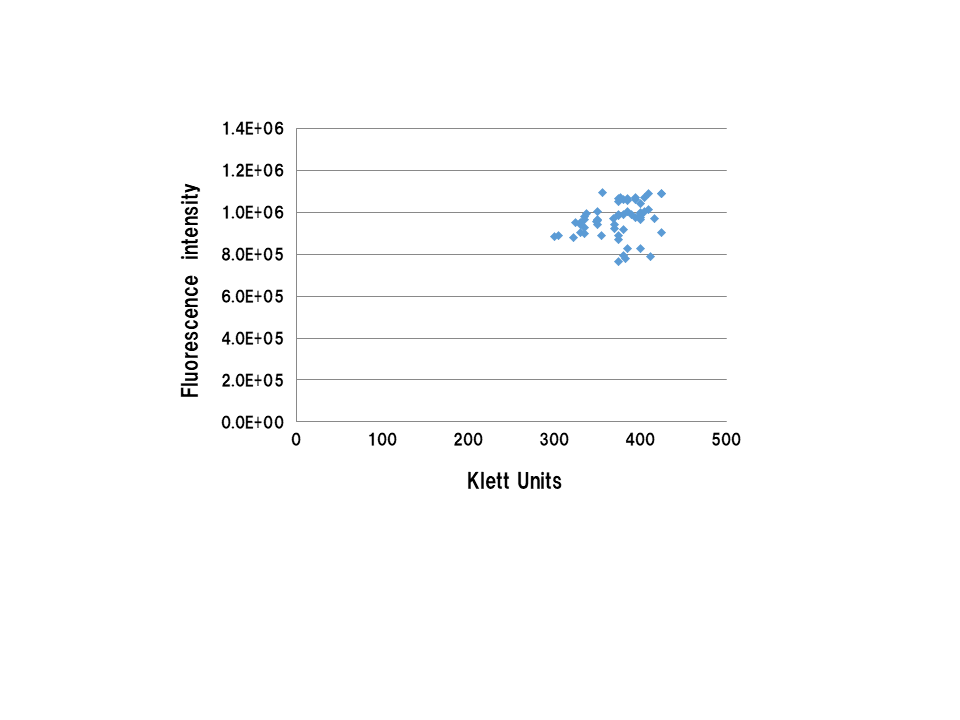

Supplement: Figure S3 — Fluorescence intensity of all tested strains. [file aem.01707-24-s0003.tif]
