# Supplementary material for: Glycophenotyping of mutants of Lacticaseibacillus paracasei by lectin microarray
Source: Appl Environ Microbiol. 2025 Jul 9;91(8):e01707-24. doi: 10.1128/aem.01707-24 (PMC12366308; doi:10.1128/aem.01707-24)
Supplement: Supplemental legends — Legends for Fig. S1 to S3. [file aem.01707-24-s0004.docx]

Fig. S1. Preparation of plasmid pRD8. Plasmid pRD8 was constructed as follows; DNA fragment-1 containing the 5’-terminal of the *CDS1932 (rmlD2)* gene were amplified with primers of RmlD1 and RmlD3 (5’-GGGGTACCTA GAGGGGAAGA GAAATGAA-3’ and 5’-CGGGATCCTA AAGTGGCGTC AATGGCC-3’). DNA fragment-1 was digested with the restriction enzymes *Kpn* I and *Bam*H I. DNA fragment-2 of the *CDS1932* (*rmlD2*) gene were amplified with primers of RmlD4 and RmlD2 (5’-CGGGATCCGA ATATGGTCAC AACTTTGTT-3’ and 5’-GCTCTAGATT ATTGCTTTTT AATCACCTGC-3’). DNA fragment-2 was digested with the restriction enzymes *Bam*H I and *Xba* I. These fragments were cloned into pBE31 (43) digested with the restriction enymes *Kpn* I and *Xba* I in the same order as on the chromosome to obtain in-frame deletions within the genes.

YIT 9029, *Lacticaseibacillus paracasei* strain Shirota; PCR, Polymerase Chain Reaction

Fig. S2. Klett Units of *L. paracasei* strain Shirota YIT 9029 and *L. casei* ATCC 334 (YIT 0180) linearly increased with culture time till 23 h at 37°C under aerobic conditions. Klett value of 100 and 200 was over 1 × 10^8^ and 1 × 10^9^ (cells/mL), respectively, for these cells. The culture time of bacterial strains was 22-24 h.

Fig. S3. The fluorescent intensity of all tested strains. The fluorescent intensity of *L. paracasei* YIT 9029, all mutants, and our other collections were labeled by SYTOX Orange, which was adjusted ≤ 2 times. Klett Units was over 300.
