# Supplementary material for: Glycophenotyping of mutants of Lacticaseibacillus paracasei by lectin microarray
Source: Appl Environ Microbiol. 2025 Jul 9;91(8):e01707-24. doi: 10.1128/aem.01707-24 (PMC12366308; doi:10.1128/aem.01707-24)
Supplement: Table S1 — Bacterial strains used in this study. [file aem.01707-24-s0005.docx]

Table S1. Bacterial strains used in this study.

| Strain or plasmid | | Relevant genotype | Source or reference |
| --- | --- | --- | --- |
| Strains | |  |  |
| *L. paracasei* strain Shirota | |  |  |
|  | YIT 9029 | Wild type | Our collection |
| 1 | Ω*0209* | Insertion in *CDS0209* gene | This study |
| 2 | Ω*0211* | Insertion in *CDS0211* gene | This study |
| 3 | Ω*0212* | Insertion in *CDS0212* gene | This study |
| 4 | Ω*0213* | Insertion in *CDS0213* gene | This study |
| 5 | Ω*0214* | Insertion in *CDS0214* gene | This study |
| 6 | Ω*0215* | Insertion in *CDS0215* gene | This study |
| 7 | Ω*0216* | Insertion in *CDS0216* gene | This study |
| 8 | Ω*0228* | Insertion in *CDS0228* gene | This study |
| 9 | Ω*0229* | Insertion in *CDS0229* gene | This study |
| 10 | Ω*0230* | Insertion in *CDS0230* gene | This study |
| 11 | Ω*0231* | Insertion in *CDS0231* gene | This study |
| 12 | Ω*0661* | Insertion in *CDS0661* gene | This study |
| 13 | Ω*0704* | Insertion in *CDS0704* gene | This study |
| 14 | Ω*0705* | Insertion in *CDS070*5 gene | This study |
| 15 | Ω*0822* | Insertion in *CDS0822* gene | This study |
| 16 | Ω*0823* | Insertion in *CDS0823* gene | This study |
| 17 | Ω*0824* | Insertion in *CDS0824* gene | This study |
| 18 | Ω*0838* | Insertion in *CDS0838* gene | This study |
| 19 | Ω*0884* | Insertion in *CDS0884* gene | This study |
| 20 | Ω*0885* | Insertion in *CDS0885* gene | This study |
| 21 | Ω*1062* | Insertion in *CDS1062* gene | This study |
| 22 | Ω*1063* | Insertion in *CDS1063* gene | This study |
| 23 | Ω*1064* | Insertion in *CDS1064* gene | This study |
| 24 | Ω*1065* | Insertion in *CDS1065* gene | This study |
| 25 | Ω*1111* | Insertion in *CDS1111* gene | This study |
| 26 | Ω*1128* | Insertion in *CDS1128* gene | This study |
| 27 | Ω*1889* | Insertion in *CDS1889* gene | This study |
| 28 | Ω*1892* | Insertion in *CDS1892* gene | This study |
| 29 | Ω*1893* | Insertion in *CDS1893* gene | This study |
| 30 | Ω*1894* | Insertion in *CDS1894* gene | This study |
| 31 | Ω*1895* | Insertion in *CDS1895* gene | This study |
| 32 | Ω*1896* | Insertion in *CDS1896* gene | This study |
| 33 | Ω*1898* | Insertion in *CDS1898* gene | This study |
| 34 | Ω*1899* | Insertion in *CDS1899* gene | This study |
| 35 | Ω*1926* | Insertion in *CDS1926* gene | This study |
| 36 | Ω*1927* | Insertion in *CDS1927* gene | This study |
| 37 | Δ*1932* | Deletion in *rmlD2* gene | This study |
| 38 | Ω*1933* | Insertion in *rmlB2* gene | This study |
| 39 | Ω*1934* | Insertion in *rmlC2* gene | This study |
| 40 | Ω*1935* | Insertion in *rmlA2* gene | This study |
| 41 | Ω*2708* | Insertion in *CDS2708* gene | This study |
| 42 | Ω*cps1A* | Insertion in *cps1A (CDS1945)* gene | (20) |
| 43 | Ω*cps1B* | Insertion in *cps1B (CDS1944)* gene | (20) |
| 44 | Δ*cps1C* | Deletion in *cps1C (CDS1943)* gene | (20,36) |
| 45 | Ω*cps1D* | Insertion in *cps1D (CDS1942)* gene | (20) |
| 46 | Ω*cps1E* | Insertion in *cps1E (CDS1941)* gene | (20) |
| 47 | Ω*cps1F* | Insertion in *cps1F (CDS1940)* gene | (20) |
| 48 | Ω*cps1G* | Insertion in *cps1G (CDS1939)* gene | (20) |
| 49 | Ω*cps1H* | Insertion in *cps1H (CDS1938*) gene | (20) |
| 50 | Ω*cps1I* | Insertion in *cps1I (CDS1937)* gene | (20) |
| 51 | Ω*cps1J* | Insertion in *cps1J (CDS1936)* gene | (20) |
| 52 | Δ*cps1A/cps1A* | Complementary of WT-*cps1A* into Δ*cps1A* | (20) |
| 53 | Δ*cps1C/cps1C* | Complementary of WT-*cps1C* into Δ*cps1C* | (20) |
| 54 | YIT 9021 | Our laboratory collection | (23) |
| 55 | YIT 9022 | Our laboratory collection | This study |
| 56 | YIT 9036 | Our laboratory collection | This study |
| 57 | YIT 9037 | Our laboratory collection | This study |
| YIT 0180^T^ | | *L. casei* neotype strain | ATCC 334 |

*L. paracasei* (formerly *L. casei*) strain Shirota (YIT 9029) is a commercial strain. The YIT number shows the culture collection preserved in the Microbiological Research Department of the Yakult Central Institute of Tokyo, Japan. Mutants were produced from YIT 9029, and Ω shows a single cross over mutant. The CDS number shows the gene that encodes the YIT 9029 protein. *L. casei ATCC* 334 is the neotype strain of *L. casei* YIT 0180, which was purchased from the American Type Culture Collection (Manassas, VA). *Escherichia coli* JM109 was purchased from Toyobo Co. Ltd. (Osaka, Japan) as competent cells for DNA transformation.
