# Supplementary material for: Glycophenotyping of mutants of Lacticaseibacillus paracasei by lectin microarray
Source: Appl Environ Microbiol. 2025 Jul 9;91(8):e01707-24. doi: 10.1128/aem.01707-24 (PMC12366308; doi:10.1128/aem.01707-24)
Supplement: Table S2 — Synthetic primers for truncated amplification. [file aem.01707-24-s0006.docx]

Table S2. Synthetic primers for truncated amplification.

| Target gene | Gene size and | Primer sequence (N- and C- terminal)  Direction* | | |
| --- | --- | --- | --- | --- |
|  |  | * | N-terminal | C-terminal |
| A gene-disrupted strains using the insertional inactivation method | | | | |
| *CDS0209* | 1452 |  | gcggatccgcagttgggccactttc | ttctgcagttagatgggactgttcaggaa |
| *CDS0211* | 747 |  | gcggatccgacgaggtgattgtgg | ttctgcagttagcggtaatacatcaattt |
| *CDS0212* | 885 |  | gcggatccgaacttgctgtcaataagc | ttctgcagttagattggccaagccattg |
| *CDS0213* | 774 |  | gcggatccgcggggtatacatttc | ttctgcagttataagccttttacccc |
| *CDS0214* | 2766 |  | ccggtaccgttagtactgtggttaacgg | gcgaattcttacttgatagcagccttatc |
| *CDS0215* | 1461 |  | ccggtaccgtactaggaccgacaggcg | gcgaattcttaaccaatcaaaacttcaacaac |
| *CDS0216* | 861 |  | cgctgcagtcgccaaatgaatcattgg | gcggatccttaataccccaacatagc |
| *CDS0228* | 759 |  | cgggatccgcggttggtgtttcacga | aactgcagggtttcagaaaccacgacc |
| *CDS0229* | 783 |  | cgggatcccagatcattgcgcatttcg | aactgcagaaccgcaccgatgatccc |
| *CDS0230* | 729 |  | cgggatccttcggcattgacaaagcttt | aactgcagggaaacatacaggcctaata |
| *CDS0231* | 333 |  | cgggatcctgggctcagtggttgctt | aactgcagggtttgaatggccatcatc |
| *CDS0661* | 762 | c | tctctgcagtatccagcaacaaacaattg | atctctagattataagcatatttgccag |
| *CDS0704* | 978 | c | ggaattcattttcgtcaatgacggctc | cgggatccttaagcgaacatcctcgc |
| *CDS0705* | 2151 | c | ggaattcccatccattcttttcgggg | cgggatccttagatgatatacggggc |
| *CDS0822* | 1209 |  | cgggatccgatcggcgaattgcggtt | aactgcagcacgacctgatgcaccat |
| *CDS0823* | 1029 |  | cgggatccggtcgcaagattggctttg | aactgcagccgcaatcgagactgcat |
| *CDS0824* | 1014 |  | cgggatcctggctgctggtggctttt | aactgcagccataacagcatccctaga |
| *CDS0838* | 1590 |  | aataagcttttagcagcgggtgac | attgctagcatcaggagactcgagac |
| *CDS0884* | 1512 |  | tataagcttggcgttgctcagacg | cactctagataaccggtttcctgatc |
| *CDS0885* | 1560 |  | atactgcagacgtttgccagccg | tactctagaatgtcatttgcgatcatc |
| *CDS1062* | 840 |  | gcgaattctaccagccattctctgcgg | ccggtaccttacttgaccgtcacaatacg |
| *CDS1063* | 957 |  | gcgaattcggcgacacaaataaactgc | ccggtaccttagtaatgccatggcttacc |
| *CDS1064* | 870 |  | gcgaattccattatttacatcagcggc | ccggtaccttaataactgacctcgcg |
| *CDS1065* | 2292 |  | gcgaattcggtgatgcgcagcaagag | ccggtaccttatagcggttgtaaggc |
| *CDS1111* | 1122 |  | cgggatccgcggcttatatgcgagaaa | aactgcagtaacacaaatagactcaggg |
| *CDS1128* | 930 | c | ttactgcagcaaatcaaacagttaca | taatctagaccattgacggccagc |
| *CDS1889* | 1545 |  | cgcgaattcctgattcaaacaactccatgg | cgggatccttactgatcaaagttgttaatgcc |
| *CDS1892* | 1059 | c | aactgcagagttacgaagggattgcct | cgggatccatgggcattgtcaatcccg |
| *CDS1893* | 843 | c | cgggatccccgtattttggccaaggcg | aactgcagggctttaccgtcacatcttta |
| *CDS1894* | 1026 | c | aactgcagacattgcagatcttcttggc | cgggatccatgacccggacgatctttc |
| *CDS1895* | 573 | c | cgggatccggcatcacaaatgattttaacc | aactgcagtaagtgatctgtcgacattgg |
| *CDS1896* | 951 | c | aactgcagctcgaagcagttggtccca | cgggatcccatagaaatacatcccggtg |
| *CDS1898* | 1401 | c | aatctgcagggctgaccatgatcacgt | acttctagaggcatacttcccgtaaac |
| *CDS1899* | 951 | c | aatatgcatttatcaatgggtctgcgg | tcatctagacctgatttaacgaggtttg |
| *CDS1926* | 765 | c | aactgcaggatgtgattcgtatgacagac | cgggatccaagtgctcgggcattctctt |
| *CDS1927* | 897 | c | cgggatcctcatataaaggtcggacagat | aactgcagtgctaagttcttaacggaacc |
| *CDS1933* | 1026 | c | cgggatccaacattgccgatttacttggt | aactgcagatgaccgggacgatccttt |
| *CDS1934* | 573 | c | cgggatccggcatcacaaatgattttaacc | aactgcagtaagtgatctgtcgacattgg |
| *CDS1935* | 933 | c | cgggatcctatcaaaacagctggttccg | aactgcaggtcataaaaatacatgccagtc |
| *CDS2708* | 1110 |  | atcctgcaggaaatgatctcgttaaaca | taatctagatattcgcgtgagccag |
| A gene-disrupted strain using the complete deletion method | | | | |
| *CDS1932　(rmlD2)* | 846 | c |  |  |
| Fragment 1 |  |  | ggggtacctagaggggaagagaaatgaa  (RmlD1 primer) | cgggatccgaatatggtcacaactttgtt  (RmlD3 primer) |
| Fragment 2 |  |  | cgggatcctaaagtggcgtcaatggcc  (RmlD4 primer) | cctctagattattgctttttaatcacctgc  (RmlD2 primer) |

Gene-disrupted strains other than *CDS1932 (rmlD2)* were created by the insertion inactivation method using one set of primers. Two sets of primers were used to disrupt *CDS1932 (rmlD2), that* was performed by the complete deletion method. The primer-positions of RmlD1, RmlD2, RmlD3, and RmlD4 were shown in Fig. S1.
