# Supplementary material for: Glycophenotyping of mutants of Lacticaseibacillus paracasei by lectin microarray
Source: Appl Environ Microbiol. 2025 Jul 9;91(8):e01707-24. doi: 10.1128/aem.01707-24 (PMC12366308; doi:10.1128/aem.01707-24)
Supplement: Table S3 — Reactivity of L. paracasei Shirota (YIT 9029) and mutants to L. paracasei Shirota-specific monoclonal antibody (MAb). [file aem.01707-24-s0007.docx]

Table S3. Reactivity of *L. paracasei* Shirota (YIT 9029) and mutants to *L. paracasei* Shirota-specific monoclonal antibody (MAb).

|  |  | Test 1 | Test 2 | Average | MAb-reactivity |
| --- | --- | --- | --- | --- | --- |
| 1 | YIT 9029 | 1.658 | 1.611 | 1.635 | Positive |
| 2 | Ω*0209* | 1.469 | 1.121 | 1.295 | Positive |
| 3 | Ω*0211* | 1.249 | 1.343 | 1.296 | Positive |
| 4 | Ω*0212* | 0.709 | 0.689 | 0.699 | Slightly positive |
| 5 | Ω*0213* | 0.852 | 0.910 | 0.881 | Slightly positive |
| 6 | Ω*0214* | 1.165 | 1.211 | 1.188 | Positive |
| 7 | Ω*0215* | 1.065 | 1.051 | 1.058 | Positive |
| 8 | Ω*0216* | 1.055 | 1.056 | 1.056 | Positive |
| 9 | Ω*0228* | 1.771 | 1.678 | 1.725 | Positive |
| 10 | Ω*0229* | 1.722 | 1.653 | 1.688 | Positive |
| 11 | Ω*0230* | 1.509 | 1.465 | 1.487 | Positive |
| 12 | Ω*0231* | 1.633 | 1.610 | 1.622 | Positive |
| 13 | Ω*0661* | 1.380 | 1.338 | 1.359 | Positive |
| 14 | Ω*0704* | 1.708 | 1.660 | 1.684 | Positive |
| 15 | Ω*0705* | 1.173 | 1.148 | 1.161 | Positive |
| 16 | Ω*0822* | 1.171 | 1.182 | 1.177 | Positive |
| 17 | Ω*0823* | 0.639 | 0.589 | 0.614 | Slightly positive |
| 18 | Ω*0824* | 0.361 | 0.486 | 0.424 | Slightly positive |
| 19 | Ω*0838* | 0.361 | 0.356 | 0.359 | Slightly positive |
| 20 | Ω*0884* | 1.251 | 1.186 | 1.219 | Positive |
| 21 | Ω*0885* | 1.425 | 1.378 | 1.402 | Positive |
| 22 | Ω*1062* | 1.781 | 1.774 | 1.778 | Positive |
| 23 | Ω*1063* | 1.734 | 1.719 | 1.727 | Positive |
| 24 | Ω*1064* | 1.732 | 1.760 | 1.746 | Positive |
| 25 | Ω*1065* | 1.548 | 1.605 | 1.577 | Positive |
| 26 | Ω*1111* | 1.336 | 1.353 | 1.345 | Positive |
| 27 | Ω*1128* | 1.464 | 1.481 | 1.473 | Positive |
| 28 | Ω*1889* | 1.088 | 1.090 | 1.089 | Positive |
| 29 | Ω*1892* | 1.779 | 1.758 | 1.769 | Positive |
| 30 | Ω*1893* | 1.653 | 1.634 | 1.644 | Positive |
| 31 | Ω*1894* | 1.642 | 1.612 | 1.627 | Positive |
| 32 | Ω*1895* | 1.541 | 1.577 | 1.559 | Positive |
| 33 | Ω*1896* | 1.529 | 1.578 | 1.554 | Positive |
| 34 | Ω*1898* | 1.472 | 1.420 | 1.446 | Positive |
| 35 | Ω*1899* | 1.508 | 1.521 | 1.515 | Positive |
| 36 | Ω*1926* | 1.401 | 1.358 | 1.380 | Positive |
| 37 | Ω*1927* | 0.897 | 0.851 | 0.874 | Slightly positive |
| 38 | Δ*1932* | 1.609 | 1.444 | 1.527 | Positive |
| 39 | Ω*1933* | 0.751 | 0.674 | 0.713 | Slightly positive |
| 40 | Ω*1934* | 1.387 | 1.292 | 1.340 | Positive |
| 41 | Ω*1935* | 1.548 | 1.471 | 1.510 | Positive |
| 42 | Ω*2708* | 1.793 | 1.767 | 1.780 | Positive |
| 43 | Ω*cps1A* | 0.162 | 0.164 | 0.163 | Negative |
| 44 | Ω*cps1B* | 0.106 | 0.108 | 0.107 | Negative |
| 45 | Δ*cps1C* | 0.112 | 0.112 | 0.112 | Negative |
| 46 | Ω*cps1D* | 0.108 | 0.099 | 0.104 | Negative |
| 47 | Ω*cps1E* | 0.130 | 0.087 | 0.109 | Negative |
| 48 | Ω*cps1F* | 0.725 | 0.690 | 0.708 | Slightly positive |
| 49 | Ω*cps1G* | 0.131 | 0.103 | 0.117 | Negative |
| 50 | Ω*cps1H* | 1.473 | 1.425 | 1.449 | Positive |
| 51 | Ω*cps1I* | 1.407 | 1.427 | 1.417 | Positive |
| 52 | Ω*cps1J* | 0.206 | 0.134 | 0.170 | Negative |
| 53 | *Δcps1C/cps1C* | 1.439 | 1.447 | 1.443 | Positive |
| 54 | YIT 9021 | 0.526 | 0.453 | 0.490 | Slightly positive |
| 55 | YIT 9022 | 1.282 | 1.241 | 1.262 | Positive |
| 56 | YIT 9036 | 0.124 | 0.108 | 0.116 | Negative |
| 57 | YIT 9037 | 0.114 | 0.088 | 0.101 | Negative |
| 58 | YIT 0180^T^　(=ATCC 334) | 0.120 | 0.090 | 0.105 | Negative |
| Blank | | 0.107 | 0.141 | 0.124 |  |

The reactivity of the mutants to YIT 9029-specific monoclonal antibody (21) was determined using a sandwich enzyme-linked immunosorbent assay, as described previously (20). The resultant fluorescence intensities of wild type and mutants were classified into three types: “positive” with full to half of the color intensity as that of wild type YIT 9029, “slightly positive” with weak or slight color intensity, and “negative” with very weak or no color. An absorbance of ≥ 1.0 was considered positive and < 0.25 was considered negative. The absorbance of ≥ 0.25 but < 1.0 was considered slightly positive. *Δcps1A/cps1A* (20) is not provided in this test.
