# Supplementary material for: Glycophenotyping of mutants of Lacticaseibacillus paracasei by lectin microarray
Source: Appl Environ Microbiol. 2025 Jul 9;91(8):e01707-24. doi: 10.1128/aem.01707-24 (PMC12366308; doi:10.1128/aem.01707-24)
Supplement: Table S4 — Sugar-binding specificities of the lectins in this study. [file aem.01707-24-s0008.docx]

**Table S4.** Sugar-binding specificities of the lectins in this study.

|  | Name | Species | Origin | Source^3^ | Rough specificity^2^ |
| --- | --- | --- | --- | --- | --- |
| 1 | LFA | *Limax flavus* | Natural | EY Lab. | Sia |
| 2 | WGA | *Triticum unlgari* | Natural | EY Lab. | (GlcNAc)n, polySia |
| 3 | PVL | *Psathyrella velutina* | Natural | Wako | Sia, GlcNAc |
| 4 | MAL | *Maackia amurensis* | Natural | Seikagaku | α2-3Sia |
| 5 | MAH | *Maackia amurensis* | Natural | Vector | α2-3Siaα2-3Sia |
| 6 | ACG | *Agrocybe cylindracea* | Natural | JOM | α2-3Sia |
| 7 | rACG | *Agrocybe cylindracea* | *E. coli* | AIST | α2-3Sia |
| 8 | rGal8N | *Homo sapiens* | *E. coli* | AIST | α2-3Sia |
| 9 | SNA | *Sambucus nigra* | Natural | Seikagaku | α2-6Sia |
| 10 | SSA | *Sambucus sieboldiana* | Natural | Vector | α2-6Sia |
| 11 | TJAI | *Trichosanthes japonica* | Natural | Vector | α2-6Sia |
| 12 | rPSL1a | *Polyporus squamosus* | *E. coli* | AIST | α2-6Sia |
| 13 | ADA | *Allomyrina dichtoma* | Natural | JOM a | 2-6Sia, Forssman, A, B |
| 14 | PHAL | *Phaseolus vulgaris* | Natural | Seikagaku | GlcNAcβ1-6Man (Tetraantenna) |
| 15 | DSA | *Datura stramonium* | Natural | Seikagaku | GlcNAcβ1-6Man (Tetraantenna) |
| 16 | TxLcI | *Tulipa gesneriana* | Natural | JOM | Galactosylated N-glycans up to triantenna |
| 17 | ECA | *Erythrina cristagalli* | Natural | Seikagaku | βGal |
| 18 | RCA120 | *Ricinus communis* | Natural | Vector | βGal |
| 19 | rGal7 | *Homo sapiens* | *E. coli* | AIST | Type 1 LacNAc, chondroitin polymer |
| 20 | rGal9N | *Homo sapiens* | *E. coli* | AIST | GalNAcα1-3Gal (A), PolyLacNAc |
| 21 | rGal9C LacNAc | *Homo sapiens* | *E. coli* | AIST | PolyLacNAc, Branched LacNAc |
| 22 | rC14 | *Gallus gallus domesticus* | *E. coli* | AIST | Branched LacNAc |
| 23 | rDiscoidin Ⅱ | *Dictyostelium Discodeum* | *E. coli* | AIST | LacNAc, Galβ1-3GalNAc (T), GalNAc (Tn) |
| 24 | BPL | *Bauhinia purpurea alba* | Natural | Vector | Galβ1-3GlcNAc(GalNAc), α/βGalNAc |
| 25 | rCGL2 | *Homo sapiens* | *E. coli* | AIST | GalNAcα1-3Gal (A), PolyLacNAc |
| 26 | PHAE | *Phaseolus vulgaris* | Natural | Vector | bisecting GlcNAc |
| 27 | GSLII | *Griffonia simplicifolia* | Natural | Vector | GlcNAcβ1-4Man |
| 28 | rSRL | *Sclerotium rolfsii* | *E. coli* | AIST | Core1,3, agalacto N-glycan |
| 29 | UDA | *Urtica dioica* | Natural | Vector | (GlcNAc)n |
| 30 | PWM | *Phytolacca americana* | Natural | Vector | (GlcNAc)n |
| 31 | rF17AG | *Escherichia coli* | *E. coli* | AIST | GlcNAc |
| 32 | rGRFT | *Griffithia sp.* | *E. coli* | AIST | Man |
| 33 | NPA | *Narcissus pseudonarcissus* | Natural | Seikagaku | Manα1-3Man |
| 34 | ConA | *Canavalia ensiformis* | Natural | Vector | Man3, Manα1-3(Manα1-6)Man）, Manα1-2Manα1-3(Manα1-6)Man, GlcNAcβ1-2Manα1-3(Manα1-6)Man |
| 35 | GNA | *Galanthus nivalis* | Natural | Vector | Manα1-3Man, Manα1-6Man |
| 36 | HHL | *Hippeastrum hybrid* | Natural | Vector | Manα1-3Man, Manα1-7Man |
| 37 | ASA | *Allium sativum* | Natural | JOM | Galβ1-4GlcNAcβ1-2Man |
| 38 | DBAI | *Dioscorea batatas* | Natural | JOM | High-man |
| 39 | CCA | *Castanea crenata* | Natural | JOM | Galactosylated N-glycans up to triantenna |
| 40 | Heltuba | *Helianthus tuberosus* | Natural | JOM | Manα1-3Man |
| 41 | rHeltuba | *Helianthus tuberosus* | *E. coli* | AIST | Manα1-3Man |
| 42 | VVAII | *Vicia villosa* | Natural | JOM | Man, Agalacto |
| 43 | rOrysata | *Oryza sativa* | *E. coli* | AIST | Manα1-3Man,  High-man, biantenna |
| 45 | rPALa | *Phlebodium aureum* | *E. coli* | AIST | Man5, biantenna |
| 45 | rBanana | *Musa acuminata* | *E. coli* | AIST | Manα1-2Manα1-3 (6) Man |
| 46 | rCalsepa | *Calystegia sepium* | *E. coli* | AIST | Biantenna with bisecting GlcNAc |
| 47 | rRSL | *Ralstonia solanacearum* | *E. coli* | AIST | αMan, α1-2Fuc (H), α1-3Fuc (Lex), α1-4Fuc (Lea) |
| 48 | rBC2LA | *Burkholderia cenocepacia* | *E. coli* | AIST | αMan, High-man |
| 49 | AOL | *Aspergillus oryzae* | Natural | Vector | α1-2Fuc (H), α1-3Fuc (Le^x^), α1-3Fuc (Le^a^) |
| 50 | AAL | *Aleuria aurantia* | Natural | Vector | α1-2Fuc (H), α1-3Fuc (Le^x^), α1-4Fuc (Lea) |
| 51 | rAAL | *Aleuria aurantia* | *E. coli* | AIST | α1-2Fuc (H), α1-3Fuc (Lex), α1-3Fuc (Lea) |
| 52 | rPAIIL | *Pseudomonas aeruginosa* | *E. coli* | AIST | αMan, α1-2Fuc (H), α1-3Fuc (Le^x^), α1-4Fuc (Le^a^) |
| 53 | rRSIIL | *Ralstonia solanacearum* | *E. coli* | AIST | α1-2Fuc (H), α1-3Fuc (Le^x^), α1-3Fuc (Le^a^) |
| 54 | rPTL | *Pholiota terrestris* | *E. coli* | AIST | α1-6Fuc |
| 55 | PSA | *Pisum sativum* | Natural | Seikagaku | α1-6Fuc up to biantenna |
| 56 | LCA | *Lens culinaris* | Natural | Vector | α1-6Fuc up to biantenna |
| 57 | rAOL | *Aspergillus oryzae* | *E. coli* | AIST | α1-2Fuc (H), α1-3Fuc (Le^x^), α1-3Fuc (Le^a^) |
| 58 | rBC2LC | *Burkholderia cenocepacia* | *E. coli* | AIST | Fucα1-2Galβ1-3GlcNAc (GalNAc) |
| 59 | LTL | *Lotus tetragonolobus* | Natural | Seikagaku | Le^x^, Le^y^ |
| 60 | UEAI | *Ulex europaeus* | Natural | Vector | α1-2Fuc |
| 61 | TJAII | *Trichosanthes japonica* | Natural | Vector | α1-2Fuc |
| 62 | MCA | *Momordica charantia* | Natural | JOM | α1-2Fuc |
| 63 | GSLI | *Griffonia simplicifolia* | Natural | Seikagaku | αGalNAc (A, Tn), αGal (B) |
| 64 | PTLI | *Psophocarpus tetragonolobu* | Natural | Tokyo Kasei | αGalNAc (A, Tn) |
| 65 | GSLIA4 | *Griffonia simplicifolia* | Natural | EY Lab. | αGalNAc (A, Tn) |
| 66 | rGC2 | *Geodia cydonium* | *E. coli* | AIST | α1-2Fuc (H), αGalNAc (A), αGal (B) |
| 67 | GSLIB4 | *Griffonia simplicifolia* | Natural | Vector | αGal (B) |
| 68 | rMOA | *Marasmius oreades* | *E. coli* | AIST | αGal (B) |
| 69 | EEL | *Euonymus europaeus* | Natural | Vector | αGal (B) |
| 70 | rPAIL | *Pseudomonas aeruginosa* | *E. coli* | AIST | α,βGal, αGalNAc (Tn) |
| 71 | LEL | *Lycopersicon esculentum* | Natural | Vector | Polylactosamine, (GlcNAc)_n_ |
| 72 | STL | *Solanum tuberosum* | Natural | Seikagaku | Polylactosamine, (GlcNAc)_n_ |
| 73 | rGal3C | *Homo sapiens* | *E. coli* | AIST | LacNAc, polylactosamine |
| 74 | rLSLN | *Laetiporus sulphureus* | *E. coli* | AIST | LacNAc, polylactosamine |
| 75 | rCGL3 | *Coprinopsis cinerea* | *E. coli* | AIST | LacDiNAc |
| 76 | PNA | *Arachis hypogaea* | Natural | Vector | Galβ1-3GalNAc (T) |
| 77 | ACA | *Amaranthus caudatus* | Natural | Vector | Galβ1-3GalNAc (T) |
| 78 | HEA | *Hericium erinaceum* | Natural | JOM | Galβ1-3GalNAc (T) |
| 79 | ABA | *Agarics bisporus* | Natural | Vector | Galβ1-3GalNAc (T), GlcNAc |
| 80 | Jacalin | *Artocarpus integrifolia* | Natural | Seikagaku | Galβ1-3GalNAc (T), GalNAcα (Tn) |
| 81 | MPA | *Maclura pomifera* | Natural | Seikagaku | Galβ1-3GalNAc (T), GalNAcα (Tn) |
| 82 | HPA | *Helix pomatia* | Natural | Seikagaku | αGalNAc (A, Tn) |
| 83 | VVA | *Vicia villosa* | Natural | Vector | α,βGalNAc (A, Tn, LacDiNAc) |
| 84 | DBA | *Dolichos biflorus* | Natural | Vector | α,βGalNAc (A, Tn, LacDiNAc) |
| 85 | SBA | *Glycine max* | Natural | EY Lab. | α,βGalNAc (A, Tn, LacDiNAc) |
| 86 | rPPL | *Pleurocybella porrigens* | *E. coli* | AIST | α,βGalNAc (A, Tn, LacDiNAc) |
| 87 | rCNL | *Clitocybe nebularis* | *E. coli* | AIST | α,βGalNAc (A, Tn, LacDiNAc) |
| 88 | rXCL | *Xerocomus chrysenteron* | *E. coli* | AIST | Core1,3, agalacto N-glycan |
| 89 | VVAⅠ | *Vicia villosa* | Natural | JOM | GalNAcβ1-3(4)Gal |
| 90 | WFA | *Wisteria floribunda* | Natural | Vector | Terminal GalNAc, LacDiNAc |
| 91 | rABA | *Agarics bisporus* | *E. coli* | AIST | Galβ1-3GalNAc (T), GlcNAc |
| 92 | rDiscoidin I | *Discodeum* *Dictyostelium* | *E. coli* | AIST | Gal |
| 93 | DBAIII | *Dioscorea batatas* | Natural | JOM | Maltose |
| 94 | rMalectin | *Homo sapiens* | *E. coli* | AIST | Glcα1-2Glc |
| 95 | CSA | *Oncorhynchus keta* | Natural | JOM | Rhamnose, Galα1-4Gal |
| 96 | rMR-Cys | *Homo sapiens* | *E. coli* | AIST | 3,4-sulfo-Gal(NAc) |

^1^Abbreviations: Gal, D-galactose; GalNAc, N-acetylgalactosamine; GlcNAc, N-acetylglucosamine; Fuc, L-fucose; Glc, D-glucose; Sia, sialic acid; LacNAc, N-acetyllactosamine.

^2^Specificity data were obtained using frontal affinity chromatography and glycoconjugate microarray.

^3^Abbreviations: JOM, J-OIL MILLS, INC; Vector, VECTOR LABORATORIES, INC; Seikagaku, SEIKAGAKU CORPORATION; EY, EY LABORATORIES, INC; and AIST, National Institute of Advanced Industrial Science and Technology.
